# Template Preparation Affects 16S rRNA High-Throughput Sequencing Analysis of Phyllosphere Microbial Communities

**DOI:** 10.3389/fpls.2017.01623

**Published:** 2017-09-26

**Authors:** Xiaoyan Tian, Yu Shi, Lili Geng, Haiyan Chu, Jie Zhang, Fuping Song, Jiangyan Duan, Changlong Shu

**Affiliations:** ^1^State Key Laboratory for Biology of Plant Diseases and Insect Pests, Institute of Plant Protection, Chinese Academy of Agricultural Sciences, Beijing, China; ^2^School of Life Sciences, Shaanxi Normal University, Linfen, China; ^3^State Key Laboratory of Soil and Sustainable Agriculture, Institute of Soil Science, Chinese Academy of Sciences, Nanjing, China

**Keywords:** phyllosphere, template, method, 16S rRNA gene, amplicon sequencing

## Abstract

Phyllosphere microbial communities are highly diverse and have important ecological implications; in that context, bacterial identification based on 16S rRNA genes is an important research issue. In studies of phyllosphere microbial communities, microporous filtration and centrifugation are used to collect microorganism samples, but it is unclear which one has a better collection efficiency. In this study, we compared these two microorganism collection methods and investigated the effects of the DNA extraction process on the estimation of microbial community composition and organization. The following four treatments were examined: (A) filtration, resuspension, and direct PCR; (B) filtration, DNA isolation, and PCR; (C) centrifugation, resuspension, and direct PCR; (D) centrifugation, DNA isolation, and PCR. Our results showed that the percentage of chloroplast sequence contaminants was affected by the DNA extraction process. The bacterial compositions clearly differed between treatments A and C, suggesting that the collection method has an influence on the determination of community structure. Compared with treatments B and D, treatments A and C resulted in higher Shannon index values, indicating that the DNA extraction process might reduce the observed phyllosphere microbial alpha diversity. However, with respect to community structure, treatments B and D yielded very similar results, suggesting that the DNA extraction process erases the effect of the collection method. Our findings provide key information to ensure accurate estimates of diversity and community composition in studies of phyllosphere microorganisms.

## Introduction

The phyllosphere is an important habitat for microbes and is colonized by a large number of microbial taxa. The global population of phyllosphere microbes is estimated to be ∼10^26^ cells (Lindow and Brandl, 2003). Culture-based isolation studies have shown that phyllosphere microbial communities include many important taxa, including plant pathogens, ice nucleation-active bacteria (Hirano and Upper, 2000), decomposers (Hicks and Silvester, 1985), phytohormone producers (Fett et al., 1987), nitrogen fixers (Furnkranz et al., 2008), and antagonists of plant pathogens (Weyman-Kaczmarkowa and Pedziwilk, 2001). Phototrophic microbes also colonize some plant leaves, including rhodopsin-based ones, absorbing different fractions of the light spectrum (Atamna-Ismaeel et al., 2012b). These findings indicate that the phyllosphere microbial community is closely related to phyllosphere functions and further studies are likely to discover new ecological interactions. Currently, both culture-dependent and culture-independent methods are used to analyze phyllosphere microorganisms. The culture-independent methods can detect a broader microbial diversity and are widely used for microbial community analyses, primarily using 16S rRNA gene amplicon sequencing to determine the composition, organization, and spatiotemporal patterns of microbial communities.

Current data indicate that bacteria occupy the majority of the plant leaf surface, ranging from 10^6^ to 10^7^ bacteria/cm^2^ (Lindow and Leveau, 2002). This concentration is lower than in other environmental samples, e.g., soil, and so a reliable and efficient bacterial collection and PCR template preparation process is needed. This process includes three main steps. First, microorganisms need to be dislodged from leaves; a sonication separation method is typically used in this step. Second, microorganisms have to be collected; two methods are used for this purpose: the microporous filtration method (Atamna-Ismaeel et al., 2012b) and the centrifugation method (Yang et al., 2001). Third, microorganism DNA is extracted using an appropriate kit (Yang et al., 2001; Atamna-Ismaeel et al., 2012b); this DNA is then used as a template for 16S rRNA gene amplicon sequencing. However, there is no consensus on the best collection method, therefore, in this study, we compared the effectiveness of different methods. Furthermore, we also investigated the effect of the DNA extraction process on estimates of microbial community composition and diversity.

The Brassicaceae are a large eudicot family and include the model plant *Arabidopsis thaliana*. *Brassica napus* L. was formed by recent allopolyploidy and diversifying selection gave rise to oilseed rape (canola), rutabaga, fodder rape, and kale morphotypes grown for oil, fodder, and food (Chalhoub et al., 2014). Analysis of the phyllosphere microbes of *B. napus* L. can not only help us understand the plant-microbe ecological interactions, but also provide ideas for *B. napus* L. insect and disease control in agriculture. In the current study, our results provide a guideline for facilitating future studies on. phyllosphere microbial communities.

## Materials and Methods

### Culture and Sample Collection

*Brassica napus* L. was selected for the analysis and the soil used to grow *B. napus* L. was collected at the Institute of Plant Protection, Chinese Academy of Agricultural Sciences. *B. napus* L. was cultured in a phytotron under the following cultivation conditions: 14 h (7000 Lux) illumination and 10 h dark, average relative humidity of 64%, temperature of 18°C to 23°C. Samples were collected in 2016 and leaves of similar sizes from three individual plants were cut from the stem and washed with phosphate-buffered saline (PBS) solution.

### Bacterial Community Collection and PCR Template Preparation

**FIGURE 1:**
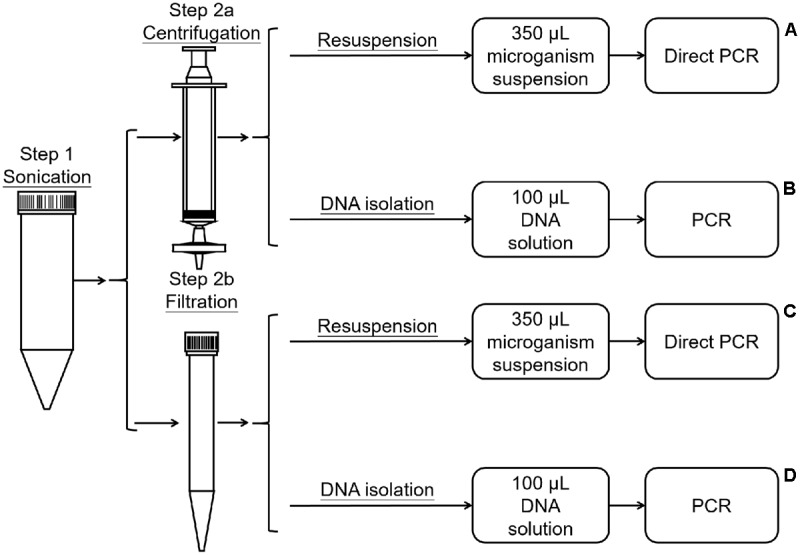
Process of bacterial collection and DNA extraction.

The method was modified from the protocols described by Yang et al. (2001) and Atamna-Ismaeel et al. (2012b). The dust on the leaves was washed off with PBS. The leaves were then placed in 50-mL tubes, submerged in 40 mL of wash buffer (0.1 M potassium phosphate buffer saline, pH 7.0), and sonicated for 10 min in an ultrasonic cleaning bath (Bransonic 32) for 10 min to dislodge bacteria (**Figure 1**, Step 1). Then, the wash buffer with dislodged microorganisms was divided into four marked A, B, C, and D. The microorganisms in A and B were collected using Millipore (0.2 μm) filtration (Billerica, MA, United States) (**Figure 1**, Step 2a), while C and D were collected by centrifugation (10,000 ×*g* for 15 min) (**Figure 1**, Step 2b). For treatments A and B, the filters were dismantled, the microorganisms in the filter membrane were resuspended in 100 μL of sterilized PBS by vortexing the minced filter membrane for 2 min, and the total microorganismal DNA in B was extracted using the Fast-DNA Kit (Axygen^®^ Corning Life Sciences, Wujiang, China) according to the manufacturer’s instructions (**Figure 1**, Step 3a, 3b). For treatments C and D, the microorganisms were resuspended in 100 μL of sterilized PBS. The microorganismal DNA in D was extracted using the Fast-DNA Kit (**Figure 1**, Step 3c, 3d). Then, 1 μL (about 3 ng) of DNA from treatment B or D was used as a template for PCR amplification, for samples A and C 1 μL of the microorganism suspension was used as a template for direct PCR. Three replicates were performed for each treatment.

### 16S rRNA Amplification and Amplicon Sequencing

The V4 hypervariable region of the 16S rRNA gene was selected for amplicon sequencing of the phyllosphere microbial community using the universal forward primer 515F (5′-GTGCCAGCMGCCGCGGTAA-3′) and the 6-bp uniquely barcoded reverse sequencing primer 806R (5′-NNNNNNGGACTACVSGGGTATCTAAT-3′) (Bokulich et al., 2012) (**Table 1**). PCR was performed as follows: 10 min of initial denaturation at 95°C, 30 cycles at 94°C for 1 min, 55°C for 1 min, and 72°C for 1 min, and a final extension at 72°C for 10 min. PCR amplicons were analyzed by 2% agarose gel electrophoresis and purified using the AxyPrep DNA Gel Extraction Kit (Axygen AP-GX-250). The purified DNA was quantified by Qubit (Thermo Fisher Scientific, United States) according to the manufacturer’s instructions. Equal amounts of PCR amplicons were mixed in a single sample. The amplicons were extended with Illumina-specific adaptors (San Diego, CA, United States) using the TruSeq^®^ DNA PCR-Free Sample Preparation Kit, and the resulting libraries were sequenced using Illumina HiSeq 2500 technology (2 × 250 bp). During this process, DNA was quantified by Qubit or quantitative PCR, as appropriate.

**Table 1:** Analysis of chloroplast contamination and OTUs based on different read counts.

Sample	Barcode	HQS	CCP %	OTUs	SRA
A1	GTTTCG	202797	90.25	284	SRR5852956
A2	CGTACG	152325	80.18	266	SRR5852952
A3	GAGTGG	167059	86.14	271	SRR5852938
B1	GGTAGC	39184	61.29	172	SRR5852944
B2	ACTGAT	161846	18.72	160	SRR5852947
B3	ATGAGC	137316	6.99	156	SRR5852933
C1	ATTCGG	75253	25.12	151	SRR5852931
C2	CAAAAG	119526	16.10	162	SRR5858743
C3	CAACTA	119719	9.74	150	SRR5858747
D1	CACCGG	151349	31.96	169	SRR5858744
D2	CACGAT	137164	5.90	154	SRR5858746
D3	CACTCA	149852	6.38	198	SRR5858745


*HQS, High-quality sequences produced from each sample. CCP, chloroplast contaminant percentage from 35000 High-quality sequences from each sample. OTUs, the operational taxonomic units produced from 7000 High-quality bacterial sequences. SRA, the accession numbers of sequence read archives deposited in NCBI*.

### Sequence Read Processing and Construction of Operational Taxonomic Unit (OTU) Tables

Before assembly, low-quality reads from the raw sequences were filtered according to the following rules: (1) reads containing only the adaptor sequence; (2) reads containing unknown nucleotides “N” in over 5% of positions; and (3) low-quality reads (defined as reads containing bases with a quality value of less than 15 occupying more than 20% of the entire read). PANDAseq was employed to correct sequence errors and merge Illumina paired-end reads to produce high-quality 16S rRNA V4 hypervariable region sequences. All processes were performed using default parameters. The UCHIME “Gold” database was used to eliminate chimeric sequences, and characteristic sequences of OTUs were selected (Edgar et al., 2011). Uparse (Usearch version 8.0.1517) (Edgar, 2013) was used to generate the 16S rRNA OTU table and determine OTU abundance. The threshold value for V4 hypervariable region identity was set to 97%. The highest frequency OTUs were determined as representative OTU sequences. The representative OTU sequences were annotated using the Ribosomal Database Project (RDP version 2.2) classifier Greengenes (version 13.8) (Wang et al., 2007).

### Downstream Data Analysis and Statistics

To assess chloroplast sequence contamination in the data sets, 35000 sequences were randomly selected for each treatment, OTU tables were constructed, and representative OTU sequences were annotated. The abundance of representative chloroplast OTUs was determined. To analyze the bacterial community composition and structure for each treatment (A, B, C, and D), data sets were normalized by randomly selecting 7000 sequences (excluding chloroplast sequences) for further analysis. The OTU table construction, OTU abundance estimation, and representative OTU sequence annotation for the 7000 bacterial sequences were performed as described before.

Alpha-diversity (i.e., within sample diversity) metrics were calculated to estimate sample community diversity using mothur (version 1.36.1) (Schloss et al., 2009). Variation between samples was calculated based on UniFrac distances, and a distance matrix was built. Weighted UniFrac distances were obtained using species abundance information and weighted branch lengths were obtained using abundance information (Lozupone and Knight, 2005). A principal coordinate analysis plot based on weighted UniFrac distances was obtained using R software (version 2.15.3) to visualize complex relationships between samples (Park et al., 2014).

## Results

### Chloroplast Sequence Contamination

After quality control and assembly, the number of high-quality sequences for each sample ranged from 39184 to 202797 (**Table 1**). To assess chloroplast sequence contamination, 35000 high-quality sequences from each treatment were randomly selected for analysis. After OTU table construction and representative OTU sequence annotation, sequence abundance data showed that treatment A resulted in an extremely high percentage (>80%) of chloroplast sequences (**Table 1**) The other samples gave a lower percentage although there was a large variation between different replicates.

### Diversity Analysis

**FIGURE 2:**
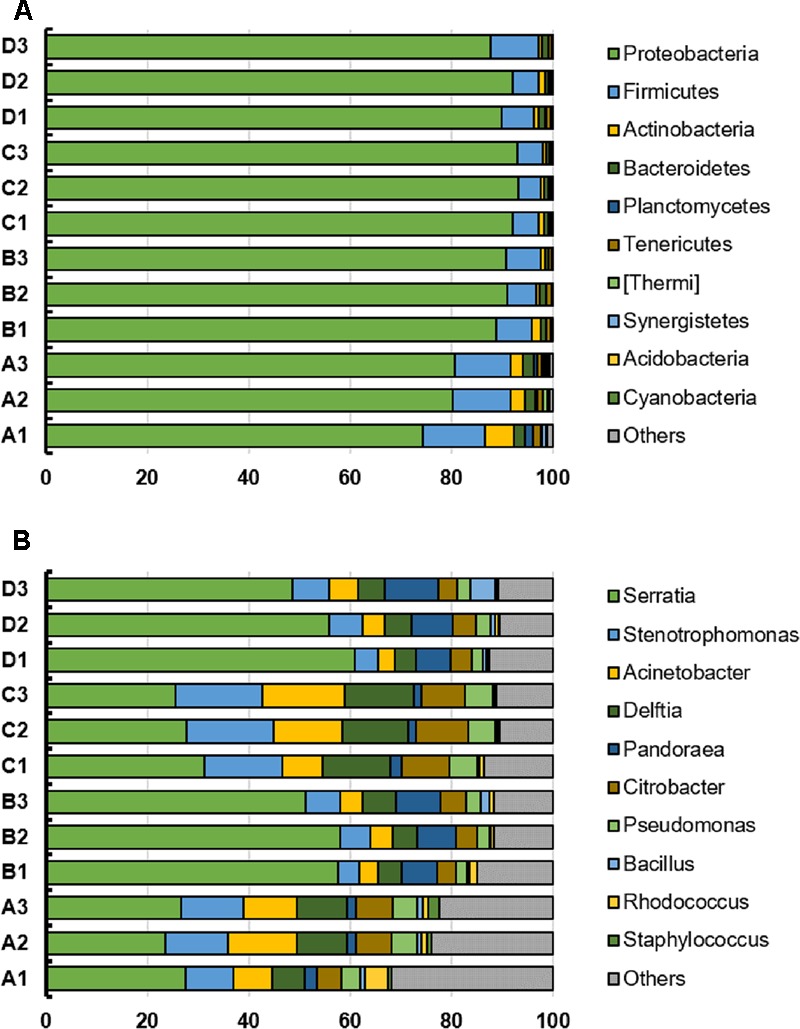
**(A,B)** Top 10 phyla and genera in individual bacterial communities from the *B. napus* L. phyllosphere for the four treatments (A, B, C, D). The *x*-axis indicates the relative abundance.

The top 10 phyla and genera based on relative abundances were compared among the four treatments (**Figure 2**). The microbial community from *B. napus* L. leaves obtained using the four treatment methods were dominated by Proteobacteria. The percentages of Proteobacteria in treatment A, B, C, and D were 78.43% (standard deviation, 2.95%), 90.23% (standard deviation, 0.97%), 92.80% (standard deviation, 0.49%) and 89.99% (standard deviation, 1.81%) respectively. At the genus level, the microbiota was dominated by *Serratia*, which was present at 25.88% for treatment A, 55.66% for treatment B, 28.22% for treatment C, and 55.10% for treatment D. *Stenotrophomonas*, *Acinetobacter*, *Delftia*, *Pandoraea*, *Citrobacter*, *Pseudomonas, Bacillus, Rhodococcus*, and *Staphylococcus* were also identified and their relative abundance was affected by template preparation method (**Figure 2**).

**FIGURE 3:**
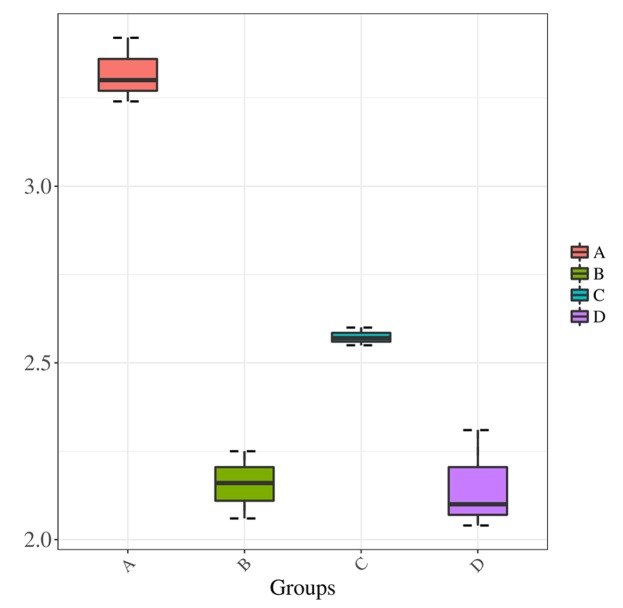
Shannon diversity estimates. Horizontal axis indicates the four treatments A, B, C, and D; vertical axis represents the Shannon diversity values.

We compared the Shannon index values obtained for samples prepared using treatments A and C, both of which used direct PCR, to determine the effect of collection method. The leaf microbiota collected by Millipore filtration (treatment A) was more diverse than that collected by centrifugation (treatment C) (*P* < 0.01) (**Figure 3**). When comparing the effect of the DNA extraction process on microbiota diversity, the Shannon index values were significantly lower for samples subjected to DNA extraction (treatments B and D) than for those subjected to direct PCR (A and C). In addition, there was no significant difference in Shannon index values between samples subjected to DNA extraction in treatments B and D. The lower Shannon index values obtained for treatments B and D compared to treatments A and C suggested that the DNA extraction process results in lower estimates of community diversity.

**FIGURE 4:**
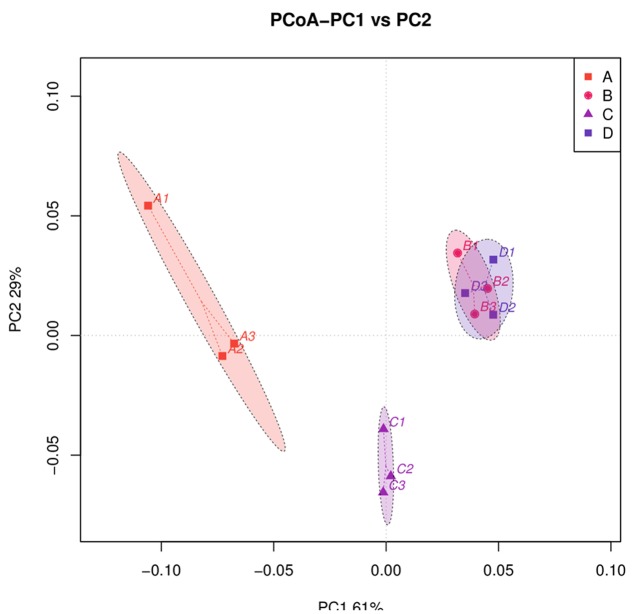
Principal coordinate analysis (PCoA) profile of pairwise community dissimilarity (Bray-Curtis) indices for 16S V4 sequencing data for the *B. napus* L. phyllosphere obtained by four different treatments (A, B, C, and D). Ellipses represent the 95% confidence intervals.

A principal coordinate analysis revealed distinct bacterial communities among the four treatments (**Figure 4**). Principal component 1 (PC1) and PC2 accounted for 60.15 and 29.37% of the community variation, respectively. Samples in the same treatment group clustered together. In particular, the bacterial communities obtained for treatments A and C were differentiated, suggesting that the community collection method affects the community structure. Additionally, bacterial community data obtained for treatments B and D were different from those for treatments A and C, indicating that the DNA extraction process could affect community structure as well. However, the bacterial community data obtained for treatments B and D could not be distinguished, and the ellipse areas, which represent the 95% confidence intervals, overlapped. These results suggest that the DNA extraction process could eliminate the differences generated by the sample collection method.

## Discussion

Green plants are primary producers that can manufacture inorganic nutrients via photosynthesis, with the leaves being the main site of photosynthesis. Previous work has demonstrated the importance of characterizing the phyllosphere microbial community, which contains many functionally important microbes. The 16S rRNA gene amplicon sequencing strategy has been successfully applied in studies of the composition, organization, and spatiotemporal patterns of microbial communities and is important to improve our understanding of phyllosphere microbial communities (Yang et al., 2001; Atamna-Ismaeel et al., 2012a). Several methods have been used for template preparation for 16S rRNA gene amplicon sequencing. In this study, we evaluated the effectiveness of the template preparation methods in 16S rRNA gene amplicon sequencing and microbial community analyses.

Normally, the first step in template preparation is to dislodge microorganisms from leaves. Separation by sonication and brush rubbing has been used for this purpose. In addition, Suda et al. (2008) attempted the direct extraction of phyllosphere microbial DNA using chemical reagents, without the dislodging step. In this study, we used sonication separation to dislodge microorganisms from leaves because the brush rubbing method is complex and not highly reproducible; furthermore, another study (Ali et al., 2015) indicated that the DNA produced using the latter method contains substantial chloroplast contamination.

We then evaluated the two main microorganism cell collection methods, i.e., microporous filtration (Atamna-Ismaeel et al., 2012b) and centrifugation (Yang et al., 2001). The microporous filtration method filters out microorganism cells that exceed the size of the pores (0.2 μm) in the filter membrane, while the centrifugation method collects microorganism cells with greater densities than that of the solution. In this study, we compared the effectiveness of the two collection methods using direct PCR (treatments A and C). The microporous filtration method resulted in a higher Shannon index value and also produced more OTUs (**Table 1**). These data suggest that the microporous filtration method is more effective for the collection of microorganisms, it did not miss microorganism cells that are not easy collected by centrifugation, e.g., microorganisms with cell densities that are not significantly greater than that of the solution.

In addition, we evaluated the effects of the DNA extraction process on microbial community analyses. DNA extraction is often necessary, e.g., in analyses of rhizosphere and intestinal microbial communities. However, the precise DNA extraction method may affect the results of these analyses. This issue has two main explanations. First, different bacterial groups (gram-negative, gram-positive, etc.) demonstrate different degrees of resistance to chemical agents or cell disruption treatments in protocols for DNA extraction (Wintzingerode et al., 1997; Nelson et al., 2010). Therefore, in some cases, bacterial cell walls cannot be broken sufficiently (depending on the composition and structure of the wall) and DNA is not released into the solution. Second, the chemical agents might lyse cells effectively, but damage DNA in the process. This leads to the underestimation and/or overestimation of some bacterial groups in the microbial community. In this study, we evaluated the effects of the PCR template preparation process on microbial community analyses (treatments B and D) by comparison with data obtained by direct PCR (treatments A and C). The DNA extraction process was associated with a lower Shannon index value. The sequence number in the OTUs table (Supplementary Table 1) also suggested that some bacterial groups as well as chloroplast DNA (**Table 1**) were not released or degraded during the extraction process while other bacterial groups were released more efficiently. With respect to the DNA extraction process, the microbial community samples obtained using different collection methods (treatment A and C) differed. When we compared the microbial community data obtained for treatments B and D, with an added DNA extraction step, with those for treatments A and C, there was no significant difference. These results suggested that the current DNA extraction method did bias the community analysis. Therefore, the DNA extraction method still needs to be improved.

## Conclusion

Based on the data obtained in this study, we suggest that microporous filtration is the preferred method for microbial cell collection to obtain a comprehensive summary of the microbial community when chloroplast contamination is acceptable. However, if it is critical to minimize chloroplast contamination, the centrifugation method is also acceptable. But, extracted DNA method is less efficient for the analysis of microbial diversity.

## Author Contributions

Design of experiment by CS, JD, YS, LG, HC, FS, JZ, and XT. Experiments performed by XT, CS, YS, and LG. First draft done of the paper by XT, and CS. Suggestions of this paper made by YS, FS, and HC.

## Conflict of Interest Statement

The authors declare that the research was conducted in the absence of any commercial or financial relationships that could be construed as a potential conflict of interest.
