# Supplementary material for: Template Preparation Affects 16S rRNA High-Throughput Sequencing Analysis of Phyllosphere Microbial Communities
Source: Front Plant Sci. 2017 Sep 26;8:1623. doi: 10.3389/fpls.2017.01623 (PMC5622981; doi:10.3389/fpls.2017.01623)
Supplement: Supplementary file 1 [file Table_1.docx]

**Template Preparation Affects16S rDNA High-throughput Sequencing Analyze of Phyllosphere** **Microbial Communities**

**Running title:** Template preparation for high-throughput sequencing

Xiaoyan Tian^1, 2^, Yu Shi^3^, Lili Geng^1^, Haiyan Chu ^3^, Jie Zhang^1^, Fuping Song ^1^, Jiangyan Duan^2*^, Changlong Shu^1*^

^1^ State Key Laboratory for Biology of Plant Diseases and Insect Pests, Institute of Plant Protection, Chinese Academy of Agricultural Sciences, Beijing, China

^2^ School of Life Sciences, Shanxi Normal University, Linfen, China

^3^State Key Laboratory of Soil and Sustainable Agriculture, Institute of Soil Science, Chinese Academy of Sciences Nanjing, China.

*Correspondence:

Changlong Shu ([clshu@ippcaas.cn](mailto:clshu@ippcaas.cn)), Jiangyan Duan (duanjiangyan123@163.com)

X.T. and Y. S. contributed equally to this work.

**Supplemental Material**

Table 1 The OTUs produced from 7000 High-quality bacteria sequences.

| OTU | A1 | A2 | A3 | B1 | B2 | B3 | C1 | C2 | C3 | D1 | D2 | D3 | Taxonomy |
| --- | --- | --- | --- | --- | --- | --- | --- | --- | --- | --- | --- | --- | --- |
| OTU_1 | 1769 | 1486 | 1684 | 3936 | 3961 | 3478 | 2002 | 1768 | 1604 | 4140 | 3794 | 3309 | k__Bacteria; p__Proteobacteria; c__Gammaproteobacteria; o__Enterobacteriales; f__Enterobacteriaceae; g__Serratia; s__marcescens |
| OTU_2 | 644 | 832 | 845 | 295 | 413 | 476 | 1066 | 1184 | 1192 | 320 | 456 | 492 | k__Bacteria; p__Proteobacteria; c__Gammaproteobacteria; o__Xanthomonadales; f__Xanthomonadaceae; g__Stenotrophomonas; s__ |
| OTU_3 | 444 | 662 | 671 | 317 | 349 | 461 | 934 | 899 | 949 | 281 | 360 | 379 | k__Bacteria; p__Proteobacteria; c__Betaproteobacteria; o__Burkholderiales; f__Comamonadaceae; g__Delftia; s__ |
| OTU_4 | 388 | 675 | 525 | 200 | 229 | 236 | 430 | 746 | 917 | 194 | 256 | 306 | k__Bacteria; p__Proteobacteria; c__Gammaproteobacteria; o__Pseudomonadales; f__Moraxellaceae; g__Acinetobacter; s__rhizosphaerae |
| OTU_5 | 168 | 124 | 120 | 482 | 529 | 601 | 142 | 103 | 100 | 481 | 564 | 727 | k__Bacteria; p__Proteobacteria; c__Betaproteobacteria; o__Burkholderiales; f__Burkholderiaceae; g__Pandoraea; s__ |
| OTU_6 | 326 | 489 | 503 | 270 | 293 | 362 | 654 | 714 | 601 | 281 | 326 | 260 | k__Bacteria; p__Proteobacteria; c__Gammaproteobacteria; o__Enterobacteriales; f__Enterobacteriaceae; g__Citrobacter; s__ |
| OTU_7 | 194 | 262 | 229 | 117 | 126 | 162 | 331 | 310 | 324 | 120 | 166 | 142 | k__Bacteria; p__Proteobacteria; c__Gammaproteobacteria; o__Pseudomonadales; f__Pseudomonadaceae; g__Pseudomonas; s__veronii |
| OTU_8 | 326 | 84 | 97 | 171 | 65 | 107 | 82 | 77 | 54 | 60 | 92 | 60 | k__Bacteria; p__Proteobacteria; c__Alphaproteobacteria; o__Rhizobiales; f__Phyllobacteriaceae; g__; s__ |
| OTU_9 | 135 | 260 | 190 | 53 | 68 | 63 | 116 | 193 | 211 | 43 | 58 | 89 | k__Bacteria; p__Proteobacteria; c__Gammaproteobacteria; o__Pseudomonadales; f__Moraxellaceae; g__Acinetobacter; s__ |
| OTU_10 | 318 | 86 | 70 | 110 | 38 | 61 | 61 | 35 | 23 | 42 | 52 | 31 | k__Bacteria; p__Actinobacteria; c__Actinobacteria; o__Actinomycetales; f__Nocardiaceae; g__Rhodococcus; s__ |
| OTU_11 | 41 | 44 | 59 | 23 | 24 | 99 | 22 | 14 | 15 | 35 | 47 | 304 | k__Bacteria; p__Firmicutes; c__Bacilli; o__Bacillales; f__Bacillaceae; g__Bacillus; s__cereus |
| OTU_12 | 82 | 65 | 50 | 37 | 27 | 53 | 143 | 62 | 99 | 25 | 35 | 33 | k__Bacteria; p__Proteobacteria; c__Alphaproteobacteria; o__Rhizobiales; f__Brucellaceae; g__Ochrobactrum; s__ |
| OTU_13 | 101 | 125 | 130 | 63 | 80 | 91 | 173 | 163 | 168 | 80 | 87 | 77 | k__Bacteria; p__Proteobacteria; c__Gammaproteobacteria; o__Enterobacteriales; f__Enterobacteriaceae; g__Serratia; s__ |
| OTU_14 | 52 | 71 | 36 | 45 | 31 | 37 | 35 | 29 | 33 | 43 | 20 | 23 | k__Bacteria; p__Firmicutes; c__Clostridia; o__Clostridiales; f__Eubacteriaceae; g__Acetobacterium; s__ |
| OTU_15 | 60 | 56 | 49 | 40 | 48 | 24 | 23 | 19 | 25 | 55 | 30 | 36 | k__Bacteria; p__Firmicutes; c__Clostridia; o__Clostridiales; f__Caldicoprobacteraceae; g__Caldicoprobacter; s__ |
| OTU_16 | 122 | 23 | 29 | 36 | 17 | 23 | 20 | 18 | 13 | 12 | 20 | 9 | k__Bacteria; p__Proteobacteria; c__Gammaproteobacteria; o__Xanthomonadales; f__Xanthomonadaceae; g__Pseudoxanthomonas |
| OTU_17 | 41 | 49 | 161 | 5 | 3 | 9 | 3 | 4 | 3 | 2 | 10 | 18 | k__Bacteria; p__Firmicutes; c__Bacilli; o__Bacillales; f__Staphylococcaceae; g__Staphylococcus; s__ |
| OTU_18 | 40 | 31 | 42 | 26 | 24 | 11 | 21 | 13 | 11 | 18 | 18 | 22 | k__Bacteria; p__Bacteroidetes; c__Bacteroidia; o__Bacteroidales; f__; g__; s__ |
| OTU_19 | 17 | 32 | 25 | 30 | 32 | 28 | 16 | 27 | 12 | 39 | 27 | 28 | k__Bacteria; p__Proteobacteria; c__Betaproteobacteria; o__Burkholderiales; f__Alcaligenaceae; g__Alcaligenes; s__faecalis |
| OTU_20 | 99 | 30 | 26 | 9 | 6 | 3 | 1 | 6 | 2 | 9 | 5 | 4 | k__Bacteria; p__Planctomycetes; c__Planctomycetia; o__Pirellulales; f__Pirellulaceae; g__; s__ |
| OTU_21 | 28 | 21 | 32 | 20 | 23 | 17 | 18 | 22 | 23 | 22 | 15 | 14 | k__Bacteria; p__Firmicutes; c__Clostridia; o__Clostridiales; f__Syntrophomonadaceae; g__Syntrophomonas; s__ |
| OTU_22 | 95 | 81 | 42 | 55 | 37 | 51 | 23 | 35 | 41 | 28 | 37 | 26 | k__Bacteria; p__Firmicutes; c__Clostridia; o__Clostridiales; f__[Tissierellaceae]; g__; s__ |
| OTU_23 | 32 | 20 | 38 | 11 | 22 | 8 | 18 | 11 | 6 | 14 | 7 | 14 | k__Bacteria; p__Bacteroidetes; c__Bacteroidia; o__Bacteroidales; f__; g__; s__ |
| OTU_24 | 29 | 26 | 24 | 13 | 12 | 14 | 19 | 19 | 20 | 14 | 13 | 10 | k__Bacteria; p__Proteobacteria; c__Gammaproteobacteria; o__Pseudomonadales; f__Pseudomonadaceae; g__Pseudomonas; s__ |
| OTU_25 | 58 | 45 | 46 | 42 | 51 | 32 | 23 | 29 | 20 | 57 | 22 | 24 | k__Bacteria; p__Tenericutes; c__Mollicutes; o__Acholeplasmatales; f__Acholeplasmataceae; g__Acholeplasma; s__ |
| OTU_26 | 12 | 4 | 9 | 9 | 14 | 21 | 12 | 3 | 24 | 18 | 17 | 30 | k__Bacteria; p__Proteobacteria; c__Betaproteobacteria; o__Burkholderiales; f__Alcaligenaceae; g__Achromobacter; s__ |
| OTU_27 | 13 | 6 | 11 | 31 | 21 | 21 | 5 | 7 | 2 | 25 | 29 | 34 | k__Bacteria; p__Proteobacteria; c__Gammaproteobacteria; o__Oceanospirillales; f__Halomonadaceae; g__Halomonas; s__ |
| OTU_28 | 26 | 29 | 20 | 18 | 17 | 9 | 14 | 8 | 9 | 23 | 15 | 18 | k__Bacteria; p__Firmicutes; c__Clostridia; o__Clostridiales; f__Clostridiaceae; g__Clostridium; s__ |
| OTU_29 | 4 | 4 | 5 | 5 | 1 | 6 | 82 | 0 | 0 | 6 | 2 | 0 | k__Bacteria; p__Proteobacteria; c__Alphaproteobacteria; o__Rhizobiales; f__Methylobacteriaceae; g__Methylobacterium; s__ |
| OTU_30 | 20 | 16 | 8 | 18 | 20 | 17 | 4 | 7 | 8 | 16 | 22 | 22 | k__Bacteria; p__Proteobacteria; c__Betaproteobacteria; o__Burkholderiales; f__Comamonadaceae; g__Hylemonella; s__ |
| OTU_31 | 21 | 35 | 45 | 19 | 16 | 18 | 41 | 46 | 41 | 12 | 15 | 21 | k__Bacteria; p__Proteobacteria; c__Alphaproteobacteria; o__Rhizobiales; f__Rhizobiaceae; g__Agrobacterium; s__ |
| OTU_32 | 19 | 12 | 10 | 10 | 18 | 6 | 10 | 5 | 4 | 16 | 9 | 14 | k__Bacteria; p__Bacteroidetes; c__Bacteroidia; o__Bacteroidales; f__Porphyromonadaceae; g__; s__ |
| OTU_33 | 7 | 12 | 11 | 3 | 8 | 4 | 28 | 15 | 21 | 4 | 7 | 5 | k__Bacteria; p__Proteobacteria; c__Alphaproteobacteria; o__Caulobacterales; f__Caulobacteraceae; g__Brevundimonas; s__diminuta |
| OTU_34 | 26 | 17 | 15 | 18 | 15 | 15 | 19 | 18 | 15 | 6 | 16 | 7 | k__Bacteria; p__Firmicutes; c__Clostridia; o__Clostridiales; f__[Tissierellaceae]; g__Gallicola; s__ |
| OTU_35 | 30 | 18 | 11 | 8 | 12 | 6 | 7 | 4 | 4 | 11 | 10 | 11 | k__Bacteria; p__Tenericutes; c__RF3; o__ML615J-28; f__; g__; s__ |
| OTU_36 | 24 | 16 | 14 | 11 | 5 | 5 | 2 | 3 | 8 | 6 | 6 | 2 | k__Bacteria; p__Firmicutes; c__Clostridia; o__SHA-98; f__; g__; s__ |
| OTU_37 | 25 | 5 | 24 | 2 | 1 | 1 | 1 | 0 | 0 | 1 | 0 | 1 | k__Bacteria; p__Proteobacteria; c__Gammaproteobacteria; o__Legionellales; f__Legionellaceae |
| OTU_38 | 10 | 10 | 4 | 5 | 3 | 6 | 3 | 6 | 6 | 8 | 0 | 9 | k__Bacteria; p__Firmicutes; c__Clostridia; o__Clostridiales; f__Clostridiaceae; g__Clostridium; s__ |
| OTU_39 | 13 | 19 | 19 | 18 | 13 | 20 | 15 | 10 | 12 | 23 | 14 | 10 | k__Bacteria; p__Firmicutes; c__Clostridia; o__Clostridiales; f__Clostridiaceae; g__Clostridium; s__ |
| OTU_40 | 36 | 15 | 9 | 1 | 0 | 1 | 3 | 1 | 0 | 2 | 2 | 0 | k__Bacteria; p__Proteobacteria; c__Betaproteobacteria; o__Burkholderiales; f__Comamonadaceae; g__Limnobacter; s__ |
| OTU_41 | 38 | 15 | 20 | 5 | 6 | 5 | 4 | 4 | 4 | 3 | 4 | 3 | k__Bacteria; p__Synergistetes; c__Synergistia; o__Synergistales; f__Dethiosulfovibrionaceae; g__Aminobacterium; s__ |
| OTU_42 | 8 | 7 | 7 | 9 | 6 | 4 | 6 | 3 | 11 | 9 | 7 | 4 | k__Bacteria; p__Firmicutes; c__Clostridia; o__Clostridiales; f__Syntrophomonadaceae; g__Syntrophomonas; s__ |
| OTU_43 | 9 | 12 | 6 | 6 | 2 | 1 | 5 | 3 | 5 | 6 | 3 | 1 | k__Bacteria; p__Firmicutes; c__Clostridia; o__Clostridiales; f__Clostridiaceae; g__02d06; s__ |
| OTU_44 | 30 | 28 | 17 | 18 | 12 | 11 | 20 | 10 | 13 | 6 | 16 | 10 | k__Bacteria; p__Firmicutes; c__Clostridia; o__Clostridiales; f__[Tissierellaceae]; g__Tissierella_Soehngenia; s__ |
| OTU_45 | 27 | 17 | 15 | 16 | 17 | 10 | 8 | 6 | 9 | 23 | 6 | 16 | k__Bacteria; p__Firmicutes; c__Clostridia; o__Clostridiales; f__[Tissierellaceae]; g__Peptoniphilus; s__ |
| OTU_46 | 4 | 5 | 12 | 3 | 2 | 11 | 0 | 1 | 5 | 3 | 2 | 31 | k__Bacteria; p__Firmicutes; c__Bacilli; o__Bacillales; f__Bacillaceae; g__Bacillus; s__ |
| OTU_47 | 7 | 8 | 3 | 4 | 0 | 3 | 4 | 6 | 1 | 1 | 5 | 2 | k__Bacteria; p__Firmicutes; c__Bacilli; o__Bacillales; f__Bacillaceae; g__Bacillus |
| OTU_48 | 16 | 14 | 7 | 7 | 2 | 4 | 10 | 3 | 9 | 9 | 4 | 3 | k__Bacteria; p__Firmicutes; c__Clostridia; o__Clostridiales; f__Clostridiaceae; g__SMB53; s__ |
| OTU_49 | 12 | 8 | 6 | 2 | 4 | 3 | 4 | 3 | 3 | 7 | 4 | 7 | k__Bacteria; p__Firmicutes; c__Clostridia; o__MBA08; f__; g__; s__ |
| OTU_50 | 14 | 15 | 4 | 3 | 3 | 1 | 10 | 4 | 9 | 3 | 4 | 3 | k__Bacteria; p__Firmicutes; c__Bacilli; o__Lactobacillales; f__Enterococcaceae; g__Vagococcus; s__ |
| OTU_51 | 9 | 19 | 30 | 0 | 0 | 0 | 0 | 0 | 1 | 0 | 1 | 0 | k__Bacteria; p__Proteobacteria; c__Gammaproteobacteria; o__Pseudomonadales; f__Moraxellaceae; g__Enhydrobacter; s__ |
| OTU_52 | 14 | 0 | 2 | 7 | 1 | 0 | 0 | 0 | 0 | 6 | 0 | 0 | k__Bacteria |
| OTU_53 | 4 | 3 | 4 | 2 | 6 | 0 | 7 | 4 | 5 | 3 | 2 | 2 | k__Bacteria; p__Firmicutes; c__Clostridia; o__Clostridiales; f__Caldicoprobacteraceae; g__Caldicoprobacter; s__ |
| OTU_54 | 7 | 12 | 20 | 0 | 0 | 0 | 0 | 0 | 2 | 0 | 3 | 7 | k__Bacteria; p__Actinobacteria; c__Actinobacteria; o__Actinomycetales; f__Micrococcaceae; g__Micrococcus; s__ |
| OTU_55 | 3 | 2 | 7 | 2 | 3 | 0 | 2 | 1 | 0 | 5 | 1 | 7 | k__Bacteria; p__Bacteroidetes; c__Bacteroidia; o__Bacteroidales; f__Porphyromonadaceae; g__; s__ |
| OTU_56 | 15 | 15 | 18 | 10 | 6 | 9 | 5 | 5 | 6 | 15 | 4 | 4 | k__Bacteria; p__Firmicutes; c__Clostridia; o__Clostridiales; f__[Tissierellaceae]; g__GW-34; s__ |
| OTU_57 | 9 | 3 | 4 | 6 | 6 | 1 | 5 | 1 | 1 | 2 | 0 | 4 | k__Bacteria; p__Firmicutes; c__Clostridia; o__Clostridiales; f__Peptococcaceae; g__; s__ |
| OTU_58 | 18 | 27 | 24 | 0 | 0 | 0 | 0 | 0 | 1 | 1 | 1 | 0 | k__Bacteria; p__[Thermi]; c__Deinococci; o__Deinococcales; f__Deinococcaceae; g__Deinococcus; s__ |
| OTU_59 | 13 | 10 | 4 | 4 | 1 | 2 | 1 | 0 | 2 | 0 | 2 | 3 | k__Bacteria; p__Firmicutes; c__Clostridia; o__SHA-98; f__; g__; s__ |
| OTU_60 | 2 | 30 | 11 | 0 | 0 | 0 | 0 | 0 | 1 | 0 | 1 | 0 | k__Bacteria; p__Proteobacteria; c__Betaproteobacteria; o__Neisseriales; f__Neisseriaceae; g__Neisseria; s__subflava |
| OTU_61 | 9 | 2 | 2 | 2 | 0 | 4 | 2 | 0 | 5 | 1 | 2 | 2 | k__Bacteria; p__Firmicutes; c__Clostridia |
| OTU_62 | 4 | 8 | 6 | 0 | 0 | 2 | 7 | 3 | 6 | 3 | 2 | 5 | k__Bacteria; p__Firmicutes; c__Bacilli; o__Lactobacillales; f__Streptococcaceae; g__Lactococcus; s__garvieae |
| OTU_63 | 7 | 4 | 4 | 3 | 1 | 0 | 5 | 3 | 7 | 3 | 8 | 5 | k__Bacteria; p__Firmicutes; c__Clostridia; o__Clostridiales; f__Syntrophomonadaceae; g__Syntrophomonas; s__ |
| OTU_64 | 7 | 10 | 4 | 4 | 6 | 6 | 4 | 6 | 5 | 5 | 3 | 3 | k__Bacteria; p__Firmicutes; c__Clostridia; o__Clostridiales; f__Clostridiaceae; g__Clostridium; s__ |
| OTU_65 | 0 | 1 | 0 | 4 | 2 | 2 | 1 | 0 | 1 | 2 | 0 | 2 | k__Bacteria; p__Firmicutes; c__Clostridia; o__Clostridiales; f__; g__; s__ |
| OTU_66 | 2 | 4 | 3 | 0 | 5 | 5 | 6 | 0 | 10 | 7 | 2 | 2 | k__Bacteria; p__Bacteroidetes; c__Sphingobacteriia; o__Sphingobacteriales; f__Sphingobacteriaceae; g__Sphingobacterium; s__mizutaii |
| OTU_67 | 8 | 4 | 15 | 4 | 4 | 7 | 6 | 6 | 3 | 2 | 4 | 7 | k__Bacteria; p__Firmicutes; c__Clostridia; o__MBA08; f__; g__; s__ |
| OTU_68 | 3 | 4 | 2 | 2 | 4 | 0 | 1 | 2 | 1 | 3 | 0 | 1 | k__Bacteria; p__Firmicutes; c__Clostridia; o__Clostridiales; f__Ruminococcaceae; g__; s__ |
| OTU_69 | 13 | 9 | 7 | 5 | 5 | 3 | 5 | 4 | 2 | 2 | 2 | 4 | k__Bacteria; p__Firmicutes; c__Clostridia; o__Clostridiales; f__Gracilibacteraceae; g__Lutispora; s__ |
| OTU_70 | 10 | 6 | 4 | 0 | 1 | 1 | 0 | 1 | 1 | 1 | 0 | 0 | k__Bacteria; p__Thermotogae; c__Thermotogae; o__Thermotogales; f__Thermotogaceae; g__AUTHM297; s__ |
| OTU_71 | 4 | 6 | 6 | 9 | 5 | 3 | 3 | 2 | 6 | 1 | 2 | 2 | k__Bacteria; p__Firmicutes; c__Clostridia; o__Clostridiales; f__[Tissierellaceae]; g__; s__ |
| OTU_72 | 7 | 2 | 2 | 2 | 2 | 2 | 5 | 4 | 4 | 4 | 1 | 7 | k__Bacteria; p__Synergistetes; c__Synergistia; o__Synergistales; f__Dethiosulfovibrionaceae; g__; s__ |
| OTU_73 | 8 | 8 | 5 | 9 | 5 | 5 | 2 | 4 | 5 | 0 | 1 | 4 | k__Bacteria; p__Firmicutes; c__Clostridia; o__Clostridiales; f__[Tissierellaceae]; g__; s__ |
| OTU_74 | 3 | 15 | 14 | 2 | 1 | 2 | 5 | 8 | 3 | 1 | 2 | 1 | k__Bacteria; p__Proteobacteria; c__Alphaproteobacteria; o__Caulobacterales; f__Caulobacteraceae; g__Mycoplana; s__ |
| OTU_75 | 3 | 4 | 2 | 2 | 1 | 1 | 1 | 0 | 2 | 2 | 2 | 1 | k__Bacteria; p__Bacteroidetes; c__Bacteroidia; o__Bacteroidales; f__; g__; s__ |
| OTU_76 | 5 | 2 | 3 | 6 | 1 | 2 | 3 | 1 | 1 | 4 | 2 | 3 | k__Bacteria; p__Proteobacteria; c__Gammaproteobacteria; o__Pseudomonadales; f__Pseudomonadaceae; g__; s__ |
| OTU_77 | 5 | 4 | 1 | 3 | 0 | 1 | 0 | 4 | 3 | 9 | 3 | 7 | k__Bacteria; p__Bacteroidetes; c__Bacteroidia; o__Bacteroidales; f__Porphyromonadaceae; g__; s__ |
| OTU_78 | 4 | 1 | 12 | 1 | 1 | 0 | 0 | 1 | 0 | 0 | 0 | 0 | k__Bacteria; p__Proteobacteria; c__Alphaproteobacteria; o__Rhodospirillales; f__Rhodospirillaceae; g__Skermanella; s__ |
| OTU_79 | 6 | 1 | 3 | 3 | 0 | 2 | 0 | 0 | 0 | 1 | 0 | 1 | k__Bacteria; p__Chloroflexi; c__Anaerolineae; o__Anaerolineales; f__Anaerolinaceae; g__T78; s__ |
| OTU_80 | 7 | 6 | 6 | 1 | 3 | 3 | 1 | 4 | 1 | 5 | 1 | 3 | k__Bacteria; p__Firmicutes; c__Clostridia; o__Clostridiales; f__[Tissierellaceae] |
| OTU_81 | 3 | 7 | 4 | 5 | 1 | 4 | 0 | 4 | 1 | 1 | 1 | 3 | k__Bacteria; p__Proteobacteria; c__Betaproteobacteria; o__Burkholderiales; f__Comamonadaceae |
| OTU_82 | 4 | 8 | 18 | 3 | 1 | 0 | 1 | 2 | 1 | 1 | 0 | 2 | k__Bacteria; p__Proteobacteria; c__Alphaproteobacteria; o__Rhodobacterales; f__Rhodobacteraceae; g__Paracoccus; s__ |
| OTU_83 | 3 | 2 | 2 | 5 | 4 | 5 | 2 | 2 | 0 | 8 | 1 | 5 | k__Bacteria; p__Firmicutes; c__Erysipelotrichi; o__Erysipelotrichales; f__Erysipelotrichaceae; g__RFN20; s__ |
| OTU_84 | 3 | 5 | 14 | 0 | 1 | 0 | 0 | 0 | 0 | 1 | 0 | 0 | k__Bacteria; p__Actinobacteria; c__Actinobacteria; o__Actinomycetales; f__Corynebacteriaceae; g__Corynebacterium; s__ |
| OTU_85 | 4 | 7 | 3 | 0 | 2 | 0 | 0 | 0 | 1 | 1 | 0 | 2 | k__Bacteria; p__Proteobacteria; c__Alphaproteobacteria; o__Rhizobiales; f__Methylobacteriaceae; g__; s__ |
| OTU_86 | 3 | 2 | 3 | 2 | 5 | 1 | 1 | 3 | 1 | 0 | 2 | 1 | k__Bacteria; p__Firmicutes; c__Bacilli; o__Bacillales; f__Planococcaceae; g__Sporosarcina; s__ |
| OTU_87 | 6 | 4 | 5 | 3 | 3 | 3 | 3 | 2 | 2 | 8 | 5 | 1 | k__Bacteria; p__Proteobacteria; c__Alphaproteobacteria; o__Rhizobiales; f__; g__; s__ |
| OTU_88 | 4 | 9 | 3 | 3 | 1 | 8 | 4 | 3 | 3 | 2 | 1 | 1 | k__Bacteria; p__Firmicutes; c__Clostridia; o__Clostridiales; f__[Tissierellaceae]; g__Tepidimicrobium; s__ |
| OTU_89 | 4 | 16 | 6 | 1 | 1 | 1 | 0 | 0 | 0 | 0 | 3 | 0 | k__Bacteria; p__Actinobacteria; c__Actinobacteria; o__Actinomycetales; f__Micrococcaceae; g__Arthrobacter; s__psychrolactophilus |
| OTU_90 | 4 | 2 | 0 | 2 | 0 | 0 | 1 | 0 | 0 | 1 | 2 | 0 | k__Bacteria; p__WS1; c__; o__; f__; g__; s__ |
| OTU_91 | 0 | 9 | 0 | 0 | 1 | 0 | 0 | 3 | 0 | 0 | 0 | 0 | k__Bacteria; p__[Thermi]; c__Deinococci; o__Thermales; f__Thermaceae; g__Thermus; s__ |
| OTU_92 | 2 | 2 | 3 | 3 | 1 | 2 | 3 | 1 | 1 | 0 | 0 | 1 | k__Bacteria; p__Firmicutes; c__Clostridia; o__Clostridiales; f__Caldicoprobacteraceae; g__Caldicoprobacter; s__ |
| OTU_93 | 9 | 18 | 13 | 0 | 0 | 2 | 0 | 1 | 0 | 1 | 0 | 0 | k__Bacteria; p__Firmicutes; c__Bacilli; o__Lactobacillales; f__Streptococcaceae; g__Streptococcus; s__ |
| OTU_94 | 11 | 3 | 5 | 2 | 2 | 2 | 1 | 1 | 5 | 3 | 3 | 4 | k__Bacteria; p__Proteobacteria; c__Alphaproteobacteria; o__Rhodobacterales; f__Rhodobacteraceae; g__Rhodobacter; s__ |
| OTU_95 | 0 | 8 | 3 | 0 | 0 | 0 | 0 | 0 | 0 | 0 | 0 | 0 | k__Bacteria; p__Bacteroidetes; c__Bacteroidia; o__Bacteroidales; f__Porphyromonadaceae; g__Porphyromonas; s__ |
| OTU_96 | 7 | 12 | 11 | 1 | 2 | 0 | 0 | 1 | 1 | 2 | 0 | 0 | k__Bacteria; p__Proteobacteria; c__Betaproteobacteria; o__Burkholderiales; f__Oxalobacteraceae; g__Janthinobacterium; s__ |
| OTU_97 | 1 | 0 | 14 | 0 | 0 | 0 | 0 | 0 | 0 | 0 | 0 | 0 | k__Bacteria; p__Cyanobacteria; c__Synechococcophycideae; o__Pseudanabaenales; f__; g__; s__ |
| OTU_98 | 7 | 14 | 12 | 0 | 2 | 0 | 1 | 1 | 1 | 2 | 1 | 1 | k__Bacteria; p__Actinobacteria; c__Actinobacteria; o__Actinomycetales; f__Intrasporangiaceae; g__Phycicoccus; s__ |
| OTU_99 | 3 | 3 | 2 | 3 | 4 | 4 | 2 | 1 | 6 | 0 | 5 | 2 | k__Bacteria; p__Firmicutes; c__Clostridia; o__Clostridiales; f__[Tissierellaceae]; g__Peptoniphilus; s__ |
| OTU_100 | 5 | 2 | 1 | 5 | 1 | 2 | 3 | 0 | 1 | 2 | 0 | 1 | k__Bacteria; p__Firmicutes; c__Clostridia; o__Clostridiales; f__[Tissierellaceae]; g__Tepidimicrobium; s__ |
| OTU_101 | 7 | 2 | 3 | 1 | 6 | 0 | 3 | 0 | 1 | 3 | 2 | 2 | k__Bacteria; p__Tenericutes; c__Mollicutes; o__Acholeplasmatales; f__Acholeplasmataceae; g__; s__ |
| OTU_102 | 0 | 5 | 2 | 0 | 0 | 0 | 0 | 0 | 0 | 0 | 1 | 2 | k__Bacteria; p__[Thermi]; c__Deinococci; o__Deinococcales; f__Deinococcaceae; g__Deinococcus; s__ |
| OTU_103 | 0 | 8 | 0 | 0 | 0 | 0 | 0 | 0 | 0 | 0 | 0 | 1 | k__Bacteria; p__[Thermi]; c__Deinococci; o__Deinococcales; f__Deinococcaceae; g__Deinococcus; s__ |
| OTU_104 | 6 | 3 | 2 | 3 | 1 | 4 | 1 | 1 | 1 | 1 | 2 | 1 | k__Bacteria; p__Firmicutes; c__Clostridia; o__Clostridiales; f__EtOH8; g__; s__ |
| OTU_105 | 1 | 1 | 1 | 0 | 3 | 1 | 1 | 2 | 1 | 3 | 0 | 2 | k__Bacteria; p__Firmicutes; c__Clostridia; o__Clostridiales; f__Ruminococcaceae; g__Clostridium |
| OTU_106 | 2 | 12 | 5 | 0 | 1 | 0 | 0 | 0 | 2 | 0 | 0 | 0 | k__Bacteria; p__Actinobacteria; c__Actinobacteria; o__Actinomycetales; f__Corynebacteriaceae; g__Corynebacterium; s__ |
| OTU_107 | 3 | 1 | 0 | 2 | 1 | 2 | 1 | 1 | 0 | 2 | 3 | 0 | k__Bacteria; p__Firmicutes; c__Clostridia; o__Natranaerobiales; f__ML1228J-1; g__; s__ |
| OTU_108 | 4 | 1 | 1 | 2 | 0 | 0 | 1 | 0 | 0 | 1 | 0 | 0 | k__Bacteria; p__Planctomycetes; c__Planctomycetia; o__Pirellulales; f__Pirellulaceae; g__; s__ |
| OTU_109 | 4 | 2 | 2 | 1 | 0 | 1 | 2 | 0 | 2 | 2 | 0 | 1 | k__Bacteria; p__Bacteroidetes; c__Bacteroidia; o__Bacteroidales; f__Porphyromonadaceae; g__; s__ |
| OTU_110 | 6 | 2 | 2 | 0 | 0 | 1 | 0 | 1 | 0 | 0 | 0 | 0 | k__Bacteria; p__Firmicutes; c__Clostridia; o__Thermoanaerobacterales; f__; g__; s__ |
| OTU_111 | 5 | 2 | 3 | 0 | 0 | 0 | 0 | 0 | 0 | 0 | 0 | 0 | k__Bacteria; p__TM6; c__SJA-4; o__; f__; g__; s__ |
| OTU_112 | 1 | 8 | 7 | 0 | 3 | 0 | 2 | 2 | 9 | 1 | 1 | 1 | k__Bacteria; p__Proteobacteria; c__Alphaproteobacteria; o__Sphingomonadales; f__Sphingomonadaceae; g__Sphingomonas; s__ |
| OTU_113 | 5 | 6 | 0 | 2 | 0 | 1 | 1 | 2 | 0 | 0 | 0 | 0 | k__Bacteria; p__Firmicutes; c__Clostridia; o__Clostridiales; f__Eubacteriaceae; g__Pseudoramibacter_Eubacterium; s__ |
| OTU_114 | 5 | 3 | 2 | 2 | 2 | 1 | 2 | 1 | 2 | 5 | 3 | 0 | k__Bacteria; p__Firmicutes; c__Clostridia; o__Clostridiales; f__[Tissierellaceae]; g__; s__ |
| OTU_115 | 6 | 5 | 1 | 0 | 0 | 2 | 0 | 1 | 2 | 3 | 4 | 1 | k__Bacteria; p__Actinobacteria; c__Actinobacteria; o__Actinomycetales; f__; g__; s__ |
| OTU_116 | 1 | 1 | 1 | 0 | 1 | 2 | 0 | 1 | 1 | 1 | 2 | 0 | k__Bacteria; p__Firmicutes; c__Clostridia; o__Thermoanaerobacterales; f__; g__; s__ |
| OTU_117 | 4 | 2 | 3 | 2 | 3 | 2 | 0 | 0 | 3 | 1 | 1 | 0 | k__Bacteria; p__Tenericutes; c__Mollicutes; o__Acholeplasmatales; f__Acholeplasmataceae; g__Acholeplasma; s__ |
| OTU_118 | 4 | 4 | 1 | 2 | 2 | 0 | 2 | 3 | 1 | 1 | 1 | 0 | k__Bacteria; p__Firmicutes; c__Clostridia; o__Clostridiales; f__[Acidaminobacteraceae]; g__Guggenheimella; s__ |
| OTU_119 | 1 | 1 | 1 | 0 | 2 | 1 | 2 | 1 | 3 | 0 | 0 | 0 | k__Bacteria; p__Bacteroidetes; c__Flavobacteriia; o__Flavobacteriales; f__[Weeksellaceae]; g__Elizabethkingia; s__meningoseptica |
| OTU_120 | 0 | 2 | 0 | 1 | 3 | 0 | 0 | 0 | 1 | 2 | 1 | 0 | k__Bacteria; p__Firmicutes; c__Clostridia; o__Clostridiales; f__Clostridiaceae; g__Clostridium; s__ |
| OTU_121 | 2 | 2 | 0 | 0 | 1 | 0 | 1 | 2 | 1 | 0 | 0 | 0 | k__Bacteria; p__Armatimonadetes; c__SJA-176; o__RB046; f__; g__; s__ |
| OTU_122 | 2 | 2 | 3 | 3 | 2 | 1 | 2 | 0 | 0 | 1 | 2 | 3 | k__Bacteria; p__Firmicutes; c__Clostridia; o__Clostridiales; f__Peptostreptococcaceae; g__Clostridium; s__sticklandii |
| OTU_123 | 0 | 6 | 1 | 0 | 0 | 0 | 0 | 1 | 0 | 0 | 0 | 0 | k__Bacteria; p__Proteobacteria; c__Gammaproteobacteria; o__Legionellales; f__Legionellaceae; g__; s__ |
| OTU_124 | 3 | 2 | 2 | 0 | 0 | 0 | 1 | 0 | 1 | 1 | 0 | 0 | k__Bacteria; p__Chloroflexi; c__Thermomicrobia; o__JG30-KF-CM45; f__; g__; s__ |
| OTU_125 | 0 | 1 | 3 | 4 | 0 | 3 | 1 | 0 | 0 | 1 | 3 | 1 | k__Bacteria; p__Firmicutes; c__Clostridia; o__Thermoanaerobacterales; f__; g__; s__ |
| OTU_126 | 3 | 3 | 1 | 2 | 1 | 0 | 1 | 1 | 1 | 0 | 1 | 2 | k__Bacteria; p__Firmicutes; c__Clostridia; o__Clostridiales; f__Lachnospiraceae; g__Coprococcus; s__ |
| OTU_127 | 8 | 2 | 4 | 2 | 5 | 2 | 2 | 2 | 1 | 2 | 0 | 3 | k__Bacteria; p__Firmicutes; c__Clostridia; o__MBA08; f__; g__; s__ |
| OTU_128 | 2 | 7 | 2 | 0 | 1 | 2 | 0 | 0 | 1 | 0 | 1 | 0 | k__Bacteria; p__Firmicutes; c__Clostridia; o__Clostridiales; f__Clostridiaceae; g__; s__ |
| OTU_129 | 6 | 2 | 3 | 0 | 0 | 1 | 0 | 3 | 1 | 0 | 0 | 1 | k__Bacteria; p__Firmicutes; c__Clostridia; o__Clostridiales; f__Clostridiaceae; g__Proteiniborus; s__ethanoligenes |
| OTU_130 | 1 | 0 | 5 | 0 | 0 | 0 | 0 | 0 | 0 | 0 | 0 | 2 | k__Bacteria; p__Proteobacteria; c__Alphaproteobacteria; o__Rickettsiales; f__Rickettsiaceae; g__Rickettsia; s__ |
| OTU_131 | 4 | 0 | 1 | 2 | 0 | 2 | 0 | 1 | 1 | 0 | 4 | 0 | k__Bacteria; p__Actinobacteria; c__Actinobacteria; o__Actinomycetales; f__Nocardiaceae; g__Rhodococcus; s__ |
| OTU_132 | 2 | 2 | 4 | 0 | 0 | 0 | 0 | 0 | 1 | 2 | 0 | 1 | k__Bacteria; p__Bacteroidetes; c__Bacteroidia; o__Bacteroidales; f__; g__; s__ |
| OTU_133 | 1 | 1 | 2 | 3 | 0 | 0 | 1 | 0 | 2 | 0 | 0 | 2 | k__Bacteria; p__Synergistetes; c__Synergistia; o__Synergistales; f__; g__; s__ |
| OTU_134 | 2 | 2 | 7 | 2 | 1 | 4 | 0 | 2 | 3 | 1 | 1 | 0 | k__Bacteria; p__Proteobacteria; c__Alphaproteobacteria; o__Rhodobacterales; f__Rhodobacteraceae; g__Paracoccus; s__ |
| OTU_135 | 2 | 1 | 0 | 2 | 1 | 0 | 2 | 0 | 0 | 0 | 0 | 1 | k__Bacteria; p__Proteobacteria; c__Deltaproteobacteria; o__Desulfobacterales; f__Desulfobulbaceae; g__Desulfobulbus; s__ |
| OTU_136 | 4 | 1 | 1 | 1 | 0 | 0 | 6 | 2 | 3 | 0 | 3 | 0 | k__Bacteria; p__Proteobacteria; c__Alphaproteobacteria; o__Rhizobiales; f__Methylobacteriaceae; g__Methylobacterium; s__organophilum |
| OTU_137 | 4 | 0 | 2 | 1 | 0 | 1 | 0 | 0 | 0 | 1 | 0 | 1 | k__Bacteria; p__Firmicutes; c__Clostridia; o__Clostridiales; f__Caldicoprobacteraceae; g__Caldicoprobacter; s__ |
| OTU_138 | 13 | 16 | 37 | 6 | 12 | 10 | 16 | 16 | 13 | 11 | 9 | 14 | k__Bacteria; p__Proteobacteria; c__Gammaproteobacteria; o__Pseudomonadales; f__Pseudomonadaceae; g__Pseudomonas; s__pseudoalcaligenes |
| OTU_139 | 4 | 3 | 1 | 1 | 0 | 0 | 1 | 0 | 0 | 0 | 0 | 0 | k__Bacteria; p__Firmicutes; c__Bacilli; o__Bacillales; f__[Exiguobacteraceae]; g__; s__ |
| OTU_140 | 11 | 26 | 25 | 7 | 6 | 13 | 30 | 18 | 25 | 10 | 12 | 12 | k__Bacteria; p__Proteobacteria; c__Gammaproteobacteria; o__Pseudomonadales; f__Pseudomonadaceae; g__Pseudomonas; s__viridiflava |
| OTU_141 | 1 | 1 | 2 | 1 | 0 | 0 | 0 | 1 | 1 | 1 | 0 | 1 | k__Bacteria; p__Actinobacteria; c__Actinobacteria; o__Bifidobacteriales; f__Bifidobacteriaceae; g__Bifidobacterium; s__adolescentis |
| OTU_142 | 1 | 3 | 1 | 1 | 1 | 1 | 2 | 2 | 1 | 2 | 0 | 1 | k__Bacteria; p__Firmicutes; c__Clostridia; o__Clostridiales; f__Clostridiaceae; g__Clostridium; s__ |
| OTU_143 | 1 | 1 | 2 | 0 | 0 | 1 | 1 | 0 | 0 | 2 | 1 | 1 | k__Bacteria; p__Firmicutes; c__Clostridia; o__Clostridiales; f__[Tissierellaceae]; g__Sedimentibacter; s__ |
| OTU_144 | 5 | 0 | 3 | 0 | 0 | 0 | 0 | 2 | 0 | 0 | 0 | 0 | k__Bacteria; p__Cyanobacteria; c__4C0d-2; o__MLE1-12; f__; g__; s__ |
| OTU_145 | 2 | 0 | 1 | 1 | 1 | 0 | 0 | 0 | 0 | 0 | 2 | 0 | k__Bacteria; p__Bacteroidetes; c__Bacteroidia; o__Bacteroidales; f__Rikenellaceae; g__Blvii28; s__ |
| OTU_146 | 3 | 0 | 2 | 1 | 1 | 2 | 1 | 0 | 0 | 1 | 4 | 3 | k__Bacteria; p__Bacteroidetes; c__Bacteroidia; o__Bacteroidales; f__Porphyromonadaceae; g__; s__ |
| OTU_147 | 4 | 0 | 1 | 1 | 0 | 0 | 1 | 1 | 0 | 2 | 1 | 0 | k__Bacteria; p__Firmicutes; c__Clostridia; o__Clostridiales |
| OTU_148 | 2 | 2 | 5 | 0 | 0 | 0 | 0 | 0 | 0 | 0 | 0 | 0 | k__Bacteria; p__[Thermi]; c__Deinococci; o__Deinococcales; f__Deinococcaceae; g__Deinococcus; s__ |
| OTU_149 | 0 | 2 | 0 | 2 | 1 | 4 | 1 | 0 | 1 | 2 | 1 | 1 | k__Bacteria; p__Proteobacteria; c__Gammaproteobacteria; o__Enterobacteriales; f__Enterobacteriaceae |
| OTU_150 | 4 | 1 | 3 | 5 | 1 | 0 | 2 | 3 | 0 | 1 | 0 | 1 | k__Bacteria; p__Synergistetes; c__Synergistia; o__Synergistales; f__Dethiosulfovibrionaceae; g__HA73; s__ |
| OTU_151 | 3 | 2 | 0 | 2 | 0 | 1 | 0 | 1 | 0 | 1 | 0 | 1 | k__Bacteria; p__Firmicutes; c__Clostridia; o__MBA08; f__; g__; s__ |
| OTU_152 | 1 | 6 | 1 | 0 | 0 | 0 | 0 | 0 | 0 | 2 | 0 | 0 | k__Bacteria; p__[Thermi]; c__Deinococci; o__Deinococcales; f__Deinococcaceae; g__Deinococcus; s__ |
| OTU_153 | 1 | 0 | 2 | 0 | 1 | 1 | 0 | 1 | 1 | 0 | 2 | 2 | k__Bacteria; p__Bacteroidetes; c__Bacteroidia; o__Bacteroidales; f__Porphyromonadaceae; g__; s__ |
| OTU_154 | 2 | 0 | 2 | 1 | 2 | 1 | 0 | 0 | 1 | 2 | 1 | 0 | k__Bacteria; p__Firmicutes; c__Clostridia; o__Clostridiales; f__Caldicoprobacteraceae; g__Caldicoprobacter; s__ |
| OTU_155 | 2 | 1 | 0 | 1 | 0 | 0 | 1 | 0 | 0 | 0 | 0 | 1 | k__Bacteria; p__Bacteroidetes; c__Bacteroidia; o__Bacteroidales; f__; g__; s__ |
| OTU_156 | 0 | 5 | 1 | 1 | 0 | 0 | 0 | 0 | 0 | 0 | 0 | 0 | k__Bacteria; p__Firmicutes; c__Bacilli; o__Lactobacillales; f__Aerococcaceae; g__Alloiococcus; s__ |
| OTU_157 | 2 | 2 | 2 | 1 | 0 | 0 | 0 | 0 | 0 | 0 | 0 | 0 | k__Bacteria; p__Firmicutes; c__Clostridia; o__Thermoanaerobacterales; f__Thermodesulfobiaceae; g__Coprothermobacter; s__ |
| OTU_158 | 1 | 2 | 3 | 1 | 1 | 0 | 0 | 1 | 1 | 1 | 2 | 1 | k__Bacteria; p__Firmicutes; c__Clostridia; o__Clostridiales; f__Clostridiaceae; g__Clostridium; s__ |
| OTU_159 | 1 | 0 | 2 | 0 | 0 | 1 | 1 | 0 | 0 | 1 | 1 | 1 | k__Bacteria; p__Firmicutes; c__Clostridia; o__Clostridiales; f__Clostridiaceae; g__Clostridium; s__ |
| OTU_160 | 3 | 1 | 7 | 1 | 0 | 0 | 0 | 1 | 0 | 0 | 1 | 1 | k__Bacteria; p__Chloroflexi; c__Ellin6529; o__; f__; g__; s__ |
| OTU_161 | 1 | 0 | 3 | 0 | 0 | 0 | 0 | 1 | 0 | 1 | 0 | 2 | k__Bacteria; p__Firmicutes; c__Clostridia; o__Clostridiales; f__Clostridiaceae; g__Clostridium; s__ |
| OTU_162 | 1 | 0 | 1 | 2 | 0 | 1 | 0 | 0 | 0 | 0 | 0 | 2 | k__Bacteria; p__Bacteroidetes; c__Bacteroidia; o__Bacteroidales; f__Marinilabiaceae; g__Ruminofilibacter; s__xylanolyticum |
| OTU_163 | 0 | 0 | 6 | 0 | 0 | 0 | 0 | 0 | 0 | 0 | 0 | 0 | k__Bacteria; p__Acidobacteria; c__Solibacteres; o__Solibacterales; f__; g__; s__ |
| OTU_164 | 3 | 0 | 1 | 0 | 0 | 1 | 0 | 3 | 0 | 0 | 0 | 0 | k__Bacteria; p__Bacteroidetes; c__Bacteroidia; o__Bacteroidales; f__; g__; s__ |
| OTU_165 | 2 | 0 | 1 | 0 | 1 | 0 | 0 | 0 | 0 | 0 | 0 | 0 | k__Bacteria; p__Firmicutes; c__Clostridia; o__OPB54; f__; g__; s__ |
| OTU_166 | 2 | 3 | 1 | 1 | 1 | 5 | 1 | 2 | 0 | 0 | 1 | 2 | k__Bacteria; p__Proteobacteria; c__Gammaproteobacteria; o__Chromatiales; f__Chromatiaceae; g__Allochromatium; s__ |
| OTU_167 | 3 | 0 | 2 | 1 | 3 | 2 | 1 | 0 | 0 | 0 | 1 | 1 | k__Bacteria; p__Firmicutes; c__Clostridia; o__Clostridiales |
| OTU_168 | 5 | 3 | 2 | 1 | 1 | 1 | 0 | 1 | 2 | 0 | 3 | 1 | k__Bacteria; p__Firmicutes; c__Clostridia; o__Clostridiales; f__Caldicoprobacteraceae; g__Caldicoprobacter; s__ |
| OTU_169 | 5 | 2 | 4 | 0 | 0 | 0 | 0 | 0 | 0 | 0 | 0 | 1 | k__Bacteria; p__Actinobacteria; c__Actinobacteria; o__Actinomycetales; f__Nocardioidaceae; g__; s__ |
| OTU_170 | 3 | 2 | 4 | 0 | 0 | 0 | 0 | 0 | 0 | 0 | 0 | 1 | k__Bacteria; p__Proteobacteria; c__Alphaproteobacteria; o__Sphingomonadales; f__Sphingomonadaceae; g__Sphingobium; s__ |
| OTU_171 | 2 | 3 | 4 | 0 | 0 | 0 | 0 | 0 | 0 | 0 | 0 | 0 | k__Bacteria; p__Proteobacteria; c__Alphaproteobacteria; o__Rhizobiales; f__Bradyrhizobiaceae; g__Balneimonas; s__ |
| OTU_172 | 0 | 7 | 0 | 0 | 0 | 0 | 0 | 0 | 0 | 0 | 0 | 0 | k__Bacteria |
| OTU_173 | 0 | 1 | 0 | 0 | 1 | 2 | 1 | 0 | 1 | 1 | 0 | 1 | k__Bacteria; p__Actinobacteria; c__Actinobacteria; o__Actinomycetales; f__Propionibacteriaceae; g__Propionibacterium; s__acnes |
| OTU_174 | 2 | 1 | 0 | 1 | 1 | 0 | 1 | 0 | 0 | 0 | 0 | 0 | k__Bacteria; p__Firmicutes; c__Bacilli; o__Bacillales; f__Planococcaceae; g__; s__ |
| OTU_175 | 3 | 3 | 3 | 1 | 2 | 2 | 2 | 2 | 0 | 0 | 2 | 1 | k__Bacteria; p__Bacteroidetes; c__Bacteroidia; o__Bacteroidales; f__Porphyromonadaceae; g__; s__ |
| OTU_176 | 0 | 5 | 1 | 0 | 0 | 1 | 0 | 0 | 0 | 0 | 0 | 0 | k__Bacteria; p__Actinobacteria; c__Actinobacteria; o__Actinomycetales; f__Pseudonocardiaceae; g__Pseudonocardia; s__ |
| OTU_177 | 0 | 3 | 2 | 0 | 0 | 0 | 1 | 0 | 0 | 0 | 0 | 0 | k__Bacteria; p__Firmicutes; c__Clostridia; o__Clostridiales; f__Lachnospiraceae; g__Shuttleworthia; s__ |
| OTU_178 | 2 | 0 | 3 | 2 | 0 | 0 | 1 | 1 | 0 | 1 | 0 | 0 | k__Bacteria; p__Bacteroidetes; c__Bacteroidia; o__Bacteroidales; f__Bacteroidaceae; g__Bacteroides; s__coprosuis |
| OTU_179 | 6 | 4 | 1 | 0 | 0 | 3 | 0 | 4 | 1 | 2 | 3 | 0 | k__Bacteria; p__Proteobacteria; c__Alphaproteobacteria; o__Rhizobiales; f__Bradyrhizobiaceae; g__Bradyrhizobium; s__ |
| OTU_180 | 2 | 0 | 1 | 1 | 1 | 0 | 0 | 1 | 0 | 0 | 0 | 1 | k__Bacteria; p__Synergistetes; c__Synergistia; o__Synergistales; f__Anaerobaculaceae; g__Anaerobaculum; s__ |
| OTU_181 | 0 | 5 | 0 | 0 | 0 | 0 | 0 | 0 | 0 | 0 | 0 | 0 | k__Bacteria; p__Bacteroidetes; c__Sphingobacteriia; o__Sphingobacteriales; f__Sphingobacteriaceae; g__; s__ |
| OTU_182 | 5 | 2 | 0 | 1 | 0 | 2 | 1 | 1 | 1 | 2 | 1 | 1 | k__Bacteria; p__Firmicutes; c__Clostridia; o__Clostridiales; f__Clostridiaceae; g__SMB53; s__ |
| OTU_183 | 2 | 2 | 1 | 0 | 0 | 1 | 0 | 0 | 0 | 1 | 0 | 0 | k__Bacteria; p__[Thermi]; c__Deinococci; o__Deinococcales; f__Deinococcaceae; g__Deinococcus; s__ |
| OTU_184 | 4 | 2 | 3 | 2 | 1 | 0 | 2 | 0 | 0 | 1 | 1 | 2 | k__Bacteria; p__Firmicutes; c__Clostridia; o__SHA-98; f__D2; g__; s__ |
| OTU_185 | 2 | 0 | 2 | 0 | 0 | 1 | 0 | 1 | 0 | 3 | 0 | 0 | k__Bacteria; p__Firmicutes; c__Clostridia; o__Clostridiales; f__Clostridiaceae; g__Clostridium; s__ |
| OTU_186 | 1 | 1 | 0 | 0 | 2 | 0 | 0 | 0 | 0 | 1 | 0 | 0 | k__Bacteria; p__Actinobacteria; c__Actinobacteria; o__Actinomycetales; f__Corynebacteriaceae; g__Corynebacterium; s__ |
| OTU_187 | 9 | 7 | 1 | 1 | 0 | 0 | 0 | 1 | 3 | 1 | 1 | 0 | k__Bacteria; p__Actinobacteria; c__Actinobacteria; o__Actinomycetales; f__Microbacteriaceae; g__Leucobacter; s__ |
| OTU_188 | 1 | 0 | 1 | 0 | 0 | 2 | 0 | 2 | 1 | 0 | 0 | 0 | k__Bacteria; p__Proteobacteria; c__Alphaproteobacteria; o__Rhodobacterales; f__Rhodobacteraceae; g__Rhodobacter; s__ |
| OTU_189 | 4 | 2 | 2 | 2 | 0 | 0 | 0 | 0 | 1 | 1 | 3 | 1 | k__Bacteria; p__Proteobacteria; c__Betaproteobacteria; o__Burkholderiales; f__Oxalobacteraceae; g__Herbaspirillum; s__ |
| OTU_190 | 3 | 1 | 0 | 0 | 0 | 0 | 1 | 1 | 0 | 1 | 0 | 0 | k__Bacteria; p__Proteobacteria; c__Alphaproteobacteria; o__Sphingomonadales; f__Erythrobacteraceae; g__; s__ |
| OTU_191 | 2 | 1 | 4 | 1 | 1 | 0 | 0 | 0 | 0 | 5 | 0 | 0 | k__Bacteria; p__Firmicutes; c__Bacilli; o__Bacillales; f__Bacillaceae; g__Bacillus; s__flexus |
| OTU_192 | 3 | 1 | 0 | 0 | 0 | 0 | 0 | 0 | 0 | 2 | 2 | 0 | k__Bacteria; p__Firmicutes; c__Clostridia; o__Clostridiales; f__Ruminococcaceae; g__Ruminococcus; s__ |
| OTU_193 | 1 | 3 | 1 | 0 | 0 | 0 | 0 | 0 | 0 | 0 | 0 | 1 | k__Bacteria; p__Actinobacteria; c__Actinobacteria; o__Actinomycetales; f__Brevibacteriaceae; g__Brevibacterium; s__ |
| OTU_194 | 1 | 2 | 1 | 1 | 0 | 1 | 0 | 0 | 0 | 2 | 0 | 0 | k__Bacteria; p__Firmicutes; c__Clostridia; o__Clostridiales; f__; g__; s__ |
| OTU_195 | 2 | 1 | 1 | 0 | 1 | 1 | 1 | 0 | 2 | 0 | 1 | 0 | k__Bacteria; p__Firmicutes; c__Clostridia; o__Clostridiales; f__[Tissierellaceae]; g__Tepidimicrobium; s__ |
| OTU_196 | 7 | 3 | 1 | 6 | 0 | 1 | 1 | 0 | 1 | 0 | 1 | 2 | k__Bacteria; p__Actinobacteria; c__Acidimicrobiia; o__Acidimicrobiales; f__Microthrixaceae; g__; s__ |
| OTU_197 | 3 | 4 | 5 | 1 | 0 | 0 | 0 | 1 | 0 | 2 | 0 | 1 | k__Bacteria; p__Actinobacteria; c__Actinobacteria; o__Actinomycetales; f__Geodermatophilaceae; g__; s__ |
| OTU_198 | 1 | 3 | 0 | 1 | 0 | 1 | 1 | 1 | 0 | 0 | 0 | 1 | k__Bacteria; p__Chloroflexi; c__Anaerolineae; o__Anaerolineales; f__Anaerolinaceae; g__T78; s__ |
| OTU_199 | 1 | 0 | 1 | 0 | 1 | 1 | 0 | 0 | 0 | 0 | 0 | 0 | k__Bacteria; p__Firmicutes; c__Clostridia; o__Clostridiales; f__Clostridiaceae; g__Clostridium; s__ |
| OTU_200 | 2 | 0 | 2 | 3 | 0 | 0 | 1 | 0 | 0 | 1 | 0 | 3 | k__Bacteria; p__Firmicutes; c__Erysipelotrichi; o__Erysipelotrichales; f__Erysipelotrichaceae; g__Erysipelothrix; s__ |
| OTU_201 | 0 | 4 | 2 | 0 | 0 | 0 | 0 | 0 | 0 | 0 | 0 | 0 | k__Bacteria; p__Proteobacteria; c__Betaproteobacteria; o__Methylophilales; f__Methylophilaceae; g__Methylobacillus; s__ |
| OTU_202 | 1 | 1 | 2 | 0 | 1 | 0 | 0 | 0 | 0 | 0 | 1 | 0 | k__Bacteria; p__Firmicutes; c__Bacilli; o__Turicibacterales; f__Turicibacteraceae; g__Turicibacter; s__ |
| OTU_203 | 0 | 3 | 0 | 0 | 0 | 0 | 0 | 0 | 0 | 1 | 2 | 0 | k__Bacteria; p__Bacteroidetes; c__Sphingobacteriia; o__Sphingobacteriales; f__Sphingobacteriaceae; g__Pedobacter; s__ |
| OTU_204 | 1 | 3 | 3 | 0 | 0 | 0 | 0 | 0 | 0 | 0 | 0 | 0 | k__Bacteria; p__Firmicutes; c__Bacilli; o__Lactobacillales; f__Lactobacillaceae; g__Lactobacillus; s__iners |
| OTU_205 | 0 | 6 | 0 | 0 | 0 | 0 | 0 | 0 | 0 | 0 | 0 | 0 | k__Bacteria; p__Bacteroidetes; c__Sphingobacteriia; o__Sphingobacteriales; f__Sphingobacteriaceae; g__Sphingobacterium; s__multivorum |
| OTU_206 | 0 | 1 | 0 | 0 | 1 | 0 | 0 | 0 | 1 | 0 | 0 | 0 | k__Bacteria; p__Firmicutes; c__Clostridia; o__Clostridiales; f__Clostridiaceae; g__Clostridium; s__ |
| OTU_207 | 1 | 1 | 2 | 0 | 1 | 0 | 1 | 0 | 1 | 0 | 0 | 0 | k__Bacteria; p__Proteobacteria; c__Deltaproteobacteria; o__Desulfovibrionales; f__Desulfovibrionaceae; g__; s__ |
| OTU_208 | 1 | 1 | 0 | 0 | 0 | 0 | 0 | 0 | 0 | 2 | 1 | 0 | k__Bacteria; p__Firmicutes; c__Clostridia; o__Clostridiales; f__[Mogibacteriaceae]; g__Mogibacterium; s__ |
| OTU_209 | 0 | 0 | 3 | 0 | 0 | 0 | 0 | 0 | 0 | 0 | 0 | 0 | k__Bacteria; p__Chloroflexi; c__Anaerolineae; o__Caldilineales; f__Caldilineaceae; g__Caldilinea; s__ |
| OTU_210 | 1 | 0 | 1 | 1 | 0 | 0 | 0 | 1 | 0 | 0 | 0 | 0 | k__Bacteria; p__Firmicutes; c__Clostridia; o__Clostridiales |
| OTU_211 | 5 | 0 | 2 | 0 | 0 | 0 | 2 | 0 | 0 | 0 | 0 | 0 | k__Bacteria; p__Proteobacteria; c__Gammaproteobacteria; o__Legionellales; f__Coxiellaceae; g__; s__ |
| OTU_212 | 0 | 2 | 0 | 0 | 3 | 0 | 0 | 0 | 0 | 0 | 0 | 0 | k__Bacteria; p__Actinobacteria; c__Actinobacteria; o__Actinomycetales; f__Actinomycetaceae; g__; s__ |
| OTU_213 | 0 | 0 | 3 | 0 | 1 | 0 | 0 | 0 | 0 | 0 | 0 | 0 | k__Bacteria; p__Firmicutes; c__Bacilli; o__Lactobacillales; f__Leuconostocaceae; g__Leuconostoc; s__ |
| OTU_214 | 1 | 3 | 3 | 0 | 0 | 0 | 0 | 0 | 0 | 0 | 4 | 0 | k__Bacteria; p__Actinobacteria; c__Actinobacteria; o__Actinomycetales; f__Cellulomonadaceae; g__Cellulomonas; s__ |
| OTU_215 | 1 | 1 | 1 | 0 | 3 | 1 | 1 | 0 | 1 | 0 | 0 | 0 | k__Bacteria; p__Proteobacteria; c__Deltaproteobacteria; o__Desulfovibrionales; f__Desulfovibrionaceae; g__Desulfovibrio; s__ |
| OTU_216 | 0 | 3 | 0 | 0 | 0 | 0 | 0 | 1 | 0 | 0 | 0 | 0 | k__Bacteria; p__Bacteroidetes; c__Cytophagia; o__Cytophagales; f__Cytophagaceae; g__Dyadobacter; s__ |
| OTU_217 | 1 | 1 | 0 | 2 | 1 | 1 | 0 | 1 | 0 | 0 | 0 | 0 | k__Bacteria; p__Proteobacteria; c__Alphaproteobacteria; o__Rhodobacterales; f__Rhodobacteraceae; g__Rhodobacter; s__ |
| OTU_218 | 0 | 1 | 2 | 0 | 0 | 1 | 1 | 0 | 0 | 0 | 0 | 2 | k__Bacteria; p__Firmicutes; c__OPB54; o__; f__; g__; s__ |
| OTU_219 | 2 | 2 | 1 | 0 | 1 | 1 | 0 | 1 | 1 | 1 | 1 | 0 | k__Bacteria; p__Firmicutes; c__Clostridia; o__Clostridiales; f__[Tissierellaceae]; g__Sedimentibacter; s__ |
| OTU_220 | 1 | 0 | 1 | 1 | 0 | 0 | 0 | 0 | 0 | 0 | 1 | 1 | k__Bacteria; p__Actinobacteria; c__Acidimicrobiia; o__Acidimicrobiales; f__Microthrixaceae; g__; s__ |
| OTU_221 | 0 | 1 | 0 | 0 | 2 | 0 | 1 | 0 | 0 | 0 | 0 | 0 | k__Bacteria; p__Actinobacteria; c__Actinobacteria; o__Actinomycetales; f__; g__; s__ |
| OTU_222 | 0 | 0 | 5 | 0 | 0 | 0 | 0 | 0 | 0 | 0 | 0 | 0 | k__Bacteria; p__Planctomycetes; c__Planctomycetia; o__Gemmatales; f__Gemmataceae; g__Gemmata; s__obscuriglobus |
| OTU_223 | 0 | 3 | 0 | 0 | 0 | 0 | 0 | 0 | 0 | 0 | 0 | 0 | k__Bacteria; p__[Thermi]; c__Deinococci; o__Deinococcales; f__Deinococcaceae; g__Deinococcus; s__ |
| OTU_224 | 2 | 0 | 0 | 1 | 0 | 0 | 0 | 0 | 0 | 0 | 0 | 1 | k__Bacteria; p__Firmicutes; c__Clostridia; o__Thermoanaerobacterales; f__; g__; s__ |
| OTU_225 | 1 | 0 | 0 | 0 | 0 | 1 | 0 | 1 | 0 | 0 | 0 | 0 | k__Bacteria |
| OTU_226 | 1 | 0 | 2 | 0 | 1 | 2 | 0 | 2 | 0 | 1 | 0 | 1 | k__Bacteria; p__Firmicutes; c__Clostridia; o__Clostridiales; f__Eubacteriaceae; g__; s__ |
| OTU_227 | 2 | 1 | 0 | 0 | 1 | 0 | 0 | 1 | 0 | 1 | 0 | 0 | k__Bacteria; p__Proteobacteria; c__Alphaproteobacteria; o__Rhodobacterales; f__Rhodobacteraceae; g__Roseivivax; s__ |
| OTU_228 | 4 | 1 | 1 | 0 | 0 | 0 | 0 | 0 | 0 | 0 | 0 | 0 | k__Bacteria; p__Proteobacteria; c__Betaproteobacteria; o__Ellin6067; f__; g__; s__ |
| OTU_229 | 0 | 1 | 0 | 0 | 0 | 0 | 0 | 0 | 0 | 0 | 0 | 2 | k__Bacteria; p__Bacteroidetes; c__[Saprospirae]; o__[Saprospirales]; f__Chitinophagaceae; g__Flavisolibacter; s__ |
| OTU_230 | 0 | 1 | 1 | 1 | 1 | 1 | 0 | 2 | 0 | 0 | 1 | 0 | k__Bacteria; p__Firmicutes; c__Clostridia; o__Clostridiales; f__Clostridiaceae; g__SMB53; s__ |
| OTU_231 | 0 | 5 | 1 | 0 | 0 | 0 | 2 | 0 | 2 | 1 | 2 | 0 | k__Bacteria; p__Bacteroidetes; c__Sphingobacteriia; o__Sphingobacteriales; f__Sphingobacteriaceae; g__Pedobacter; s__ |
| OTU_232 | 1 | 0 | 4 | 0 | 0 | 0 | 0 | 0 | 2 | 0 | 0 | 0 | k__Bacteria; p__Actinobacteria; c__Acidimicrobiia; o__Acidimicrobiales; f__; g__; s__ |
| OTU_233 | 1 | 0 | 2 | 0 | 0 | 0 | 0 | 0 | 1 | 0 | 0 | 0 | k__Bacteria; p__Proteobacteria; c__Gammaproteobacteria; o__Alteromonadales; f__Alteromonadaceae; g__Cellvibrio; s__ |
| OTU_234 | 1 | 1 | 0 | 0 | 0 | 0 | 0 | 1 | 0 | 0 | 0 | 0 | k__Bacteria; p__Firmicutes; c__Clostridia; o__Clostridiales; f__Gracilibacteraceae; g__Lutispora; s__ |
| OTU_235 | 2 | 1 | 0 | 0 | 1 | 0 | 0 | 0 | 0 | 0 | 1 | 0 | k__Bacteria; p__Proteobacteria; c__Alphaproteobacteria; o__Rhizobiales; f__Hyphomicrobiaceae; g__Rhodoplanes; s__ |
| OTU_236 | 0 | 0 | 2 | 1 | 0 | 0 | 0 | 0 | 1 | 0 | 2 | 0 | k__Bacteria; p__Firmicutes; c__Clostridia; o__Clostridiales; f__Ruminococcaceae; g__Ruminococcus; s__ |
| OTU_237 | 0 | 0 | 0 | 3 | 0 | 0 | 1 | 1 | 0 | 0 | 2 | 0 | k__Bacteria; p__Firmicutes; c__Clostridia; o__Clostridiales; f__Caldicoprobacteraceae; g__Caldicoprobacter; s__ |
| OTU_238 | 4 | 2 | 0 | 0 | 0 | 0 | 0 | 0 | 0 | 0 | 0 | 0 | k__Bacteria; p__Cyanobacteria; c__Synechococcophycideae; o__Pseudanabaenales; f__Pseudanabaenaceae; g__Pseudanabaena; s__galeata |
| OTU_239 | 2 | 2 | 0 | 0 | 0 | 1 | 1 | 0 | 0 | 0 | 2 | 3 | k__Bacteria; p__Firmicutes; c__Clostridia; o__Clostridiales; f__Syntrophomonadaceae; g__Syntrophomonas; s__ |
| OTU_240 | 1 | 2 | 1 | 0 | 0 | 1 | 0 | 0 | 0 | 0 | 0 | 0 | k__Bacteria; p__Actinobacteria; c__Actinobacteria; o__Actinomycetales; f__Microbacteriaceae; g__Rathayibacter; s__ |
| OTU_241 | 2 | 0 | 1 | 0 | 0 | 0 | 0 | 0 | 0 | 0 | 0 | 0 | k__Bacteria; p__Fusobacteria; c__Fusobacteriia; o__Fusobacteriales; f__Leptotrichiaceae; g__Leptotrichia; s__ |
| OTU_242 | 0 | 0 | 0 | 0 | 0 | 2 | 2 | 1 | 0 | 0 | 0 | 0 | k__Bacteria; p__Firmicutes; c__Clostridia; o__Clostridiales; f__Clostridiaceae; g__Clostridium; s__ |
| OTU_243 | 2 | 1 | 1 | 0 | 0 | 0 | 0 | 0 | 0 | 0 | 0 | 0 | k__Bacteria; p__Bacteroidetes; c__[Saprospirae]; o__[Saprospirales]; f__Chitinophagaceae; g__Flavisolibacter; s__ |
| OTU_244 | 0 | 0 | 1 | 0 | 0 | 0 | 0 | 1 | 0 | 0 | 0 | 0 | k__Bacteria; p__Proteobacteria; c__Gammaproteobacteria; o__Alteromonadales; f__Shewanellaceae; g__Shewanella; s__ |
| OTU_245 | 0 | 0 | 2 | 0 | 0 | 1 | 0 | 0 | 1 | 0 | 0 | 0 | k__Bacteria; p__Proteobacteria; c__Gammaproteobacteria; o__Aeromonadales; f__Aeromonadaceae; g__; s__ |
| OTU_246 | 0 | 0 | 2 | 0 | 0 | 0 | 0 | 0 | 0 | 0 | 0 | 0 | k__Bacteria; p__Proteobacteria; c__Alphaproteobacteria; o__Rhodospirillales; f__Acetobacteraceae; g__; s__ |
| OTU_247 | 1 | 5 | 1 | 0 | 0 | 0 | 0 | 0 | 0 | 0 | 0 | 0 | k__Bacteria; p__Fusobacteria; c__Fusobacteriia; o__Fusobacteriales; f__Fusobacteriaceae; g__Fusobacterium; s__ |
| OTU_248 | 1 | 2 | 3 | 0 | 0 | 0 | 0 | 0 | 0 | 0 | 0 | 0 | k__Bacteria; p__Firmicutes; c__Bacilli; o__Lactobacillales; f__Lactobacillaceae; g__Lactobacillus |
| OTU_249 | 2 | 1 | 0 | 0 | 0 | 0 | 0 | 1 | 0 | 0 | 0 | 0 | k__Bacteria; p__Firmicutes; c__Bacilli; o__Bacillales; f__Alicyclobacillaceae; g__Alicyclobacillus; s__ |
| OTU_250 | 3 | 0 | 1 | 0 | 0 | 0 | 0 | 0 | 0 | 1 | 0 | 0 | k__Bacteria; p__Proteobacteria; c__Betaproteobacteria; o__Methylophilales; f__Methylophilaceae; g__Methylotenera; s__mobilis |
| OTU_251 | 0 | 1 | 0 | 0 | 0 | 0 | 0 | 1 | 1 | 0 | 0 | 1 | k__Bacteria; p__Proteobacteria; c__Betaproteobacteria; o__Burkholderiales; f__Alcaligenaceae; g__; s__ |
| OTU_252 | 0 | 2 | 0 | 0 | 0 | 1 | 0 | 0 | 0 | 0 | 0 | 0 | k__Bacteria; p__Planctomycetes; c__Planctomycetia; o__Gemmatales; f__Gemmataceae; g__Gemmata; s__ |
| OTU_253 | 0 | 3 | 2 | 0 | 0 | 0 | 0 | 0 | 0 | 0 | 0 | 0 | k__Bacteria; p__Proteobacteria; c__Alphaproteobacteria; o__Rhizobiales; f__Phyllobacteriaceae; g__Mesorhizobium; s__ |
| OTU_254 | 1 | 1 | 2 | 0 | 0 | 0 | 0 | 1 | 0 | 0 | 1 | 1 | k__Bacteria; p__Firmicutes; c__Clostridia; o__Clostridiales; f__Caldicoprobacteraceae; g__Caldicoprobacter; s__ |
| OTU_255 | 0 | 2 | 0 | 0 | 0 | 0 | 0 | 0 | 0 | 0 | 0 | 0 | k__Bacteria; p__Proteobacteria; c__Alphaproteobacteria; o__Rhizobiales; f__Methylobacteriaceae; g__; s__ |
| OTU_256 | 0 | 0 | 0 | 0 | 0 | 0 | 0 | 2 | 0 | 0 | 0 | 0 | k__Bacteria; p__Proteobacteria; c__Alphaproteobacteria; o__Rhizobiales; f__Methylocystaceae; g__Pleomorphomonas; s__ |
| OTU_257 | 0 | 0 | 0 | 0 | 0 | 0 | 0 | 0 | 0 | 0 | 1 | 2 | k__Bacteria; p__Firmicutes; c__Clostridia |
| OTU_258 | 2 | 0 | 4 | 0 | 0 | 0 | 0 | 0 | 0 | 1 | 0 | 0 | k__Bacteria; p__Chloroflexi; c__Thermomicrobia; o__JG30-KF-CM45; f__; g__; s__ |
| OTU_259 | 0 | 2 | 0 | 0 | 0 | 0 | 0 | 0 | 0 | 1 | 1 | 1 | k__Bacteria; p__Bacteroidetes; c__Bacteroidia; o__Bacteroidales; f__Porphyromonadaceae; g__Paludibacter; s__ |
| OTU_260 | 0 | 0 | 2 | 0 | 0 | 0 | 0 | 0 | 0 | 0 | 0 | 0 | k__Bacteria; p__Planctomycetes; c__Planctomycetia; o__Gemmatales; f__Gemmataceae; g__; s__ |
| OTU_261 | 0 | 0 | 0 | 0 | 0 | 0 | 0 | 1 | 0 | 1 | 0 | 0 | k__Bacteria; p__Proteobacteria; c__Alphaproteobacteria; o__Rhizobiales; f__; g__; s__ |
| OTU_262 | 0 | 1 | 0 | 0 | 0 | 1 | 0 | 0 | 1 | 0 | 0 | 0 | k__Bacteria; p__Firmicutes; c__Clostridia; o__Clostridiales |
| OTU_263 | 1 | 0 | 0 | 1 | 0 | 1 | 0 | 2 | 0 | 0 | 0 | 0 | k__Bacteria; p__Proteobacteria; c__Alphaproteobacteria; o__Caulobacterales; f__Caulobacteraceae; g__Mycoplana; s__ |
| OTU_264 | 5 | 1 | 2 | 0 | 0 | 0 | 0 | 0 | 0 | 0 | 0 | 0 | k__Bacteria; p__Firmicutes; c__Clostridia; o__Clostridiales; f__[Tissierellaceae]; g__Anaerococcus; s__ |
| OTU_265 | 0 | 0 | 2 | 0 | 0 | 0 | 0 | 0 | 0 | 0 | 0 | 0 | k__Bacteria; p__Planctomycetes; c__Planctomycetia; o__Gemmatales; f__Gemmataceae; g__; s__ |
| OTU_266 | 1 | 2 | 3 | 0 | 1 | 0 | 0 | 0 | 0 | 0 | 0 | 0 | k__Bacteria; p__Acidobacteria; c__[Chloracidobacteria]; o__RB41; f__Ellin6075; g__; s__ |
| OTU_267 | 0 | 0 | 0 | 2 | 0 | 0 | 0 | 0 | 0 | 0 | 0 | 0 | k__Bacteria; p__Actinobacteria; c__Coriobacteriia; o__Coriobacteriales; f__Coriobacteriaceae; g__; s__ |
| OTU_268 | 1 | 2 | 0 | 2 | 0 | 1 | 0 | 0 | 2 | 2 | 0 | 2 | k__Bacteria; p__Firmicutes; c__Clostridia; o__MBA08; f__; g__; s__ |
| OTU_269 | 2 | 0 | 4 | 0 | 0 | 0 | 0 | 0 | 0 | 0 | 0 | 0 | k__Bacteria; p__Acidobacteria; c__Acidobacteria-6; o__iii1-15; f__mb2424; g__; s__ |
| OTU_270 | 0 | 0 | 1 | 1 | 1 | 0 | 0 | 2 | 0 | 0 | 0 | 0 | k__Bacteria; p__Proteobacteria; c__Alphaproteobacteria; o__Rhodobacterales; f__Rhodobacteraceae; g__Paracoccus; s__aminovorans |
| OTU_271 | 0 | 0 | 2 | 0 | 0 | 1 | 0 | 1 | 0 | 0 | 0 | 0 | k__Bacteria; p__Spirochaetes; c__Spirochaetes; o__Sphaerochaetales; f__Sphaerochaetaceae; g__Sphaerochaeta; s__ |
| OTU_272 | 1 | 0 | 0 | 0 | 0 | 0 | 1 | 0 | 0 | 0 | 0 | 0 | k__Bacteria; p__Firmicutes; c__Clostridia; o__Clostridiales; f__Clostridiaceae; g__Clostridium; s__ |
| OTU_273 | 0 | 4 | 3 | 0 | 0 | 0 | 0 | 0 | 0 | 0 | 0 | 0 | k__Bacteria; p__Firmicutes; c__Bacilli; o__Lactobacillales; f__Lactobacillaceae; g__Lactobacillus; s__ |
| OTU_274 | 2 | 0 | 0 | 0 | 0 | 0 | 0 | 0 | 0 | 0 | 0 | 0 | k__Bacteria; p__Proteobacteria; c__Alphaproteobacteria; o__Rhodospirillales; f__Acetobacteraceae |
| OTU_275 | 1 | 2 | 1 | 0 | 0 | 0 | 0 | 0 | 0 | 1 | 0 | 0 | k__Bacteria; p__Proteobacteria; c__Gammaproteobacteria; o__Xanthomonadales; f__Xanthomonadaceae; g__Thermomonas; s__ |
| OTU_276 | 2 | 0 | 0 | 0 | 0 | 0 | 1 | 0 | 0 | 0 | 0 | 0 | k__Bacteria; p__Proteobacteria; c__Alphaproteobacteria; o__Caulobacterales; f__Caulobacteraceae; g__Caulobacter; s__ |
| OTU_277 | 1 | 2 | 3 | 0 | 0 | 0 | 0 | 0 | 0 | 0 | 0 | 0 | k__Bacteria; p__Acidobacteria; c__[Chloracidobacteria]; o__RB41; f__Ellin6075; g__; s__ |
| OTU_278 | 1 | 0 | 0 | 1 | 0 | 0 | 0 | 0 | 0 | 0 | 0 | 0 | k__Bacteria; p__Firmicutes; c__Clostridia; o__Clostridiales; f__Ruminococcaceae; g__; s__ |
| OTU_279 | 4 | 1 | 1 | 0 | 0 | 0 | 0 | 0 | 0 | 0 | 0 | 0 | k__Bacteria; p__Actinobacteria; c__Actinobacteria; o__Actinomycetales; f__Brevibacteriaceae; g__Brevibacterium; s__aureum |
| OTU_280 | 3 | 1 | 0 | 0 | 0 | 0 | 0 | 0 | 0 | 0 | 0 | 0 | k__Bacteria; p__Bacteroidetes; c__Cytophagia; o__Cytophagales; f__Cytophagaceae; g__Hymenobacter; s__ |
| OTU_281 | 2 | 1 | 1 | 0 | 0 | 0 | 0 | 0 | 0 | 0 | 0 | 0 | k__Bacteria; p__Proteobacteria; c__Alphaproteobacteria; o__Rhizobiales; f__Rhizobiaceae; g__Agrobacterium; s__ |
| OTU_282 | 2 | 2 | 0 | 0 | 0 | 0 | 0 | 0 | 0 | 0 | 0 | 1 | k__Bacteria; p__Actinobacteria; c__Actinobacteria; o__Actinomycetales; f__Corynebacteriaceae; g__Corynebacterium; s__ |
| OTU_283 | 0 | 1 | 0 | 2 | 1 | 2 | 1 | 0 | 0 | 0 | 0 | 1 | k__Bacteria; p__Firmicutes; c__Erysipelotrichi; o__Erysipelotrichales; f__Erysipelotrichaceae; g__L7A_E11; s__ |
| OTU_284 | 1 | 1 | 0 | 0 | 0 | 0 | 0 | 0 | 0 | 0 | 0 | 0 | k__Bacteria; p__Firmicutes; c__Clostridia; o__Clostridiales; f__Ruminococcaceae; g__Faecalibacterium; s__prausnitzii |
| OTU_285 | 1 | 0 | 0 | 2 | 0 | 0 | 0 | 1 | 0 | 0 | 0 | 0 | k__Bacteria; p__Actinobacteria; c__Actinobacteria; o__Actinomycetales; f__Actinomycetaceae; g__Actinomyces; s__ |
| OTU_286 | 0 | 0 | 0 | 1 | 0 | 0 | 0 | 1 | 0 | 0 | 0 | 0 | k__Bacteria; p__Bacteroidetes; c__Bacteroidia; o__Bacteroidales; f__; g__; s__ |
| OTU_287 | 0 | 0 | 2 | 0 | 0 | 0 | 0 | 0 | 0 | 0 | 0 | 0 | k__Bacteria; p__Acidobacteria; c__Acidobacteria-6; o__iii1-15; f__mb2424; g__; s__ |
| OTU_288 | 1 | 0 | 1 | 0 | 0 | 0 | 0 | 2 | 0 | 0 | 0 | 0 | k__Bacteria; p__Firmicutes; c__Clostridia; o__Clostridiales; f__Ruminococcaceae; g__; s__ |
| OTU_289 | 3 | 0 | 1 | 0 | 0 | 0 | 0 | 0 | 0 | 0 | 0 | 0 | k__Bacteria; p__Proteobacteria; c__Betaproteobacteria; o__MND1; f__; g__; s__ |
| OTU_290 | 4 | 0 | 0 | 0 | 0 | 0 | 0 | 0 | 0 | 1 | 0 | 0 | k__Bacteria; p__Bacteroidetes; c__Cytophagia; o__Cytophagales; f__Cytophagaceae; g__Hymenobacter; s__ |
| OTU_291 | 1 | 1 | 5 | 0 | 0 | 0 | 0 | 0 | 0 | 0 | 0 | 0 | k__Bacteria; p__Actinobacteria; c__Actinobacteria; o__Actinomycetales; f__Dermabacteraceae; g__Brachybacterium; s__ |
| OTU_292 | 4 | 3 | 3 | 0 | 0 | 1 | 0 | 0 | 0 | 1 | 1 | 1 | k__Bacteria; p__Proteobacteria; c__Betaproteobacteria; o__Burkholderiales; f__Oxalobacteraceae; g__Ralstonia; s__ |
| OTU_293 | 0 | 0 | 1 | 0 | 0 | 0 | 0 | 0 | 0 | 0 | 1 | 0 | k__Bacteria; p__Chloroflexi; c__Thermomicrobia; o__JG30-KF-CM45; f__; g__; s__ |
| OTU_294 | 0 | 1 | 0 | 0 | 1 | 0 | 0 | 0 | 0 | 0 | 0 | 0 | k__Bacteria; p__Firmicutes; c__Clostridia; o__Clostridiales; f__Caldicoprobacteraceae; g__Caldicoprobacter; s__ |
| OTU_295 | 2 | 0 | 0 | 0 | 0 | 0 | 0 | 0 | 1 | 0 | 0 | 0 | k__Bacteria; p__Firmicutes; c__Clostridia; o__Clostridiales; f__[Tissierellaceae]; g__Gallicola; s__ |
| OTU_296 | 1 | 0 | 0 | 0 | 0 | 1 | 0 | 0 | 0 | 0 | 0 | 0 | k__Bacteria; p__Firmicutes; c__Clostridia; o__MBA08; f__; g__; s__ |
| OTU_297 | 3 | 1 | 0 | 2 | 0 | 0 | 1 | 1 | 0 | 1 | 0 | 0 | k__Bacteria; p__Synergistetes; c__Synergistia; o__Synergistales; f__Synergistaceae; g__vadinCA02; s__ |
| OTU_298 | 1 | 0 | 0 | 1 | 0 | 0 | 0 | 0 | 0 | 0 | 1 | 0 | k__Bacteria; p__Actinobacteria; c__Actinobacteria; o__Actinomycetales; f__Microbacteriaceae; g__Agromyces; s__ |
| OTU_299 | 1 | 0 | 0 | 0 | 1 | 0 | 0 | 0 | 1 | 0 | 0 | 0 | k__Bacteria; p__Firmicutes; c__Clostridia; o__MBA08; f__; g__; s__ |
| OTU_300 | 2 | 0 | 0 | 0 | 0 | 0 | 0 | 0 | 0 | 0 | 0 | 0 | k__Bacteria; p__Gemmatimonadetes; c__Gemmatimonadetes; o__Gemmatimonadales; f__; g__; s__ |
| OTU_301 | 0 | 0 | 0 | 0 | 1 | 1 | 0 | 1 | 0 | 2 | 0 | 0 | k__Bacteria; p__Bacteroidetes; c__Bacteroidia; o__Bacteroidales; f__Bacteroidaceae; g__BF311; s__ |
| OTU_302 | 0 | 1 | 0 | 1 | 1 | 1 | 1 | 1 | 1 | 0 | 0 | 0 | k__Bacteria; p__Verrucomicrobia; c__Verruco-5; o__WCHB1-41; f__WCHB1-25; g__; s__ |
| OTU_303 | 0 | 2 | 2 | 0 | 0 | 0 | 0 | 0 | 0 | 0 | 0 | 0 | k__Bacteria; p__Acidobacteria; c__Acidobacteria-6; o__iii1-15; f__mb2424; g__; s__ |
| OTU_304 | 0 | 0 | 3 | 0 | 0 | 0 | 0 | 0 | 0 | 0 | 1 | 0 | k__Bacteria; p__Bacteroidetes; c__Flavobacteriia; o__Flavobacteriales; f__[Weeksellaceae]; g__Chryseobacterium; s__ |
| OTU_305 | 2 | 6 | 3 | 0 | 0 | 0 | 0 | 0 | 0 | 1 | 1 | 1 | k__Bacteria; p__Actinobacteria; c__Actinobacteria; o__Actinomycetales; f__Nocardioidaceae; g__; s__ |
| OTU_306 | 0 | 1 | 0 | 1 | 0 | 0 | 0 | 0 | 0 | 0 | 0 | 1 | k__Bacteria; p__Proteobacteria; c__Gammaproteobacteria; o__Pseudomonadales; f__Moraxellaceae; g__Psychrobacter; s__pulmonis |
| OTU_307 | 1 | 0 | 0 | 1 | 0 | 1 | 0 | 0 | 2 | 0 | 0 | 0 | k__Bacteria; p__Firmicutes; c__Clostridia; o__Clostridiales; f__; g__; s__ |
| OTU_308 | 0 | 0 | 0 | 0 | 0 | 0 | 0 | 1 | 1 | 0 | 0 | 0 | k__Bacteria; p__Proteobacteria; c__Gammaproteobacteria; o__Xanthomonadales; f__Xanthomonadaceae; g__Stenotrophomonas; s__ |
| OTU_309 | 0 | 0 | 1 | 1 | 1 | 0 | 0 | 0 | 1 | 0 | 0 | 0 | k__Bacteria; p__Proteobacteria; c__Alphaproteobacteria; o__Rhizobiales; f__; g__; s__ |
| OTU_310 | 1 | 1 | 2 | 0 | 0 | 0 | 0 | 0 | 0 | 0 | 0 | 0 | k__Bacteria; p__Verrucomicrobia; c__[Pedosphaerae]; o__[Pedosphaerales]; f__Ellin517; g__; s__ |
| OTU_311 | 1 | 0 | 2 | 0 | 0 | 0 | 0 | 0 | 0 | 0 | 0 | 0 | k__Bacteria; p__Nitrospirae; c__Nitrospira; o__Nitrospirales; f__0319-6A21; g__; s__ |
| OTU_312 | 0 | 1 | 0 | 0 | 0 | 0 | 0 | 0 | 0 | 1 | 0 | 0 | k__Bacteria; p__Proteobacteria; c__Alphaproteobacteria; o__Rhodospirillales; f__Acetobacteraceae; g__; s__ |
| OTU_313 | 2 | 0 | 0 | 0 | 0 | 0 | 0 | 0 | 0 | 0 | 0 | 0 | k__Bacteria; p__Acidobacteria; c__Solibacteres; o__Solibacterales; f__Solibacteraceae; g__Candidatus Solibacter; s__ |
| OTU_314 | 0 | 2 | 1 | 0 | 0 | 0 | 0 | 0 | 0 | 0 | 0 | 0 | k__Bacteria; p__Firmicutes; c__Clostridia; o__Clostridiales; f__[Tissierellaceae]; g__Peptoniphilus; s__ |
| OTU_315 | 3 | 3 | 2 | 0 | 1 | 0 | 0 | 0 | 0 | 0 | 0 | 1 | k__Bacteria; p__Firmicutes; c__Bacilli; o__Bacillales; f__Bacillaceae; g__Bacillus |
| OTU_316 | 0 | 3 | 0 | 0 | 0 | 0 | 0 | 0 | 0 | 0 | 0 | 0 | k__Bacteria; p__Proteobacteria; c__Gammaproteobacteria; o__Chromatiales; f__Ectothiorhodospiraceae; g__; s__ |
| OTU_317 | 5 | 0 | 0 | 0 | 0 | 0 | 1 | 2 | 0 | 1 | 2 | 1 | k__Bacteria; p__Firmicutes; c__Clostridia; o__Clostridiales; f__Ruminococcaceae; g__; s__ |
| OTU_318 | 1 | 1 | 1 | 0 | 0 | 0 | 0 | 0 | 0 | 0 | 0 | 0 | k__Bacteria; p__Actinobacteria; c__Thermoleophilia; o__Gaiellales; f__Gaiellaceae; g__; s__ |
| OTU_319 | 1 | 0 | 0 | 0 | 1 | 0 | 0 | 0 | 0 | 0 | 1 | 1 | k__Bacteria; p__Proteobacteria; c__Alphaproteobacteria; o__Rhizobiales; f__Xanthobacteraceae; g__Xanthobacter; s__ |
| OTU_320 | 2 | 2 | 1 | 2 | 1 | 3 | 1 | 1 | 1 | 2 | 0 | 0 | k__Bacteria; p__Firmicutes; c__Clostridia; o__Clostridiales; f__[Acidaminobacteraceae]; g__Guggenheimella; s__ |
| OTU_321 | 0 | 5 | 0 | 0 | 0 | 0 | 0 | 0 | 0 | 0 | 0 | 0 | k__Bacteria; p__[Thermi]; c__Deinococci; o__Deinococcales; f__Deinococcaceae; g__Deinococcus; s__ |
| OTU_322 | 0 | 0 | 3 | 1 | 0 | 0 | 0 | 0 | 0 | 0 | 0 | 0 | k__Bacteria; p__Proteobacteria; c__Gammaproteobacteria; o__Pasteurellales; f__Pasteurellaceae; g__Haemophilus; s__parainfluenzae |
| OTU_323 | 2 | 5 | 10 | 3 | 6 | 2 | 3 | 4 | 1 | 6 | 1 | 7 | k__Bacteria; p__Bacteroidetes; c__Bacteroidia; o__Bacteroidales; f__; g__; s__ |
| OTU_324 | 0 | 0 | 2 | 0 | 0 | 0 | 0 | 0 | 0 | 0 | 0 | 0 | k__Bacteria; p__Cyanobacteria; c__Oscillatoriophycideae; o__Chroococcales; f__Xenococcaceae; g__; s__ |
| OTU_325 | 0 | 0 | 2 | 0 | 0 | 0 | 0 | 0 | 0 | 0 | 0 | 0 | k__Bacteria; p__Planctomycetes; c__Planctomycetia; o__Gemmatales; f__Gemmataceae; g__Gemmata; s__ |
| OTU_326 | 0 | 1 | 1 | 2 | 1 | 0 | 0 | 0 | 0 | 0 | 0 | 0 | k__Bacteria; p__Bacteroidetes; c__Bacteroidia; o__Bacteroidales; f__; g__; s__ |
| OTU_327 | 0 | 0 | 1 | 0 | 0 | 0 | 0 | 0 | 0 | 0 | 0 | 1 | k__Bacteria; p__Bacteroidetes; c__Bacteroidia; o__Bacteroidales; f__Porphyromonadaceae; g__Porphyromonas; s__endodontalis |
| OTU_328 | 0 | 0 | 3 | 0 | 0 | 0 | 0 | 0 | 0 | 0 | 0 | 0 | k__Bacteria; p__Actinobacteria; c__Actinobacteria; o__Actinomycetales; f__Micrococcaceae |
| OTU_329 | 0 | 0 | 1 | 1 | 1 | 0 | 1 | 0 | 0 | 0 | 1 | 0 | k__Bacteria; p__Firmicutes; c__Clostridia; o__Clostridiales; f__Ruminococcaceae; g__Oscillospira; s__ |
| OTU_330 | 0 | 0 | 1 | 0 | 0 | 0 | 1 | 0 | 0 | 0 | 0 | 0 | k__Bacteria; p__Firmicutes; c__Clostridia; o__Clostridiales; f__Ruminococcaceae; g__Butyricicoccus; s__pullicaecorum |
| OTU_331 | 1 | 1 | 0 | 0 | 0 | 0 | 0 | 0 | 0 | 1 | 0 | 0 | k__Bacteria; p__Firmicutes; c__Clostridia; o__Clostridiales; f__Ruminococcaceae |
| OTU_332 | 1 | 1 | 1 | 0 | 0 | 0 | 0 | 0 | 0 | 0 | 0 | 0 | k__Bacteria; p__Proteobacteria; c__Gammaproteobacteria; o__Xanthomonadales; f__Sinobacteraceae; g__Steroidobacter; s__ |
| OTU_333 | 9 | 10 | 11 | 8 | 5 | 13 | 16 | 23 | 21 | 13 | 11 | 12 | k__Bacteria; p__Proteobacteria; c__Gammaproteobacteria; o__Enterobacteriales; f__Enterobacteriaceae; g__Escherichia; s__coli |
| OTU_334 | 3 | 1 | 0 | 0 | 0 | 0 | 0 | 0 | 0 | 0 | 0 | 0 | k__Bacteria; p__Proteobacteria; c__Alphaproteobacteria; o__Rhodospirillales; f__Rhodospirillaceae; g__; s__ |
| OTU_335 | 0 | 0 | 4 | 0 | 0 | 0 | 0 | 0 | 0 | 0 | 0 | 0 | k__Bacteria; p__Acidobacteria; c__Acidobacteria-6; o__iii1-15; f__; g__; s__ |
| OTU_336 | 2 | 0 | 0 | 0 | 0 | 0 | 0 | 0 | 0 | 0 | 0 | 0 | k__Bacteria; p__Proteobacteria; c__Gammaproteobacteria; o__HTCC2188; f__HTCC2089; g__; s__ |
| OTU_337 | 0 | 0 | 0 | 0 | 1 | 0 | 1 | 0 | 0 | 0 | 0 | 0 | k__Bacteria; p__Firmicutes; c__Clostridia; o__Clostridiales; f__Clostridiaceae; g__Clostridium; s__ |
| OTU_338 | 0 | 1 | 0 | 0 | 0 | 0 | 0 | 0 | 1 | 1 | 1 | 0 | k__Bacteria; p__Proteobacteria; c__Alphaproteobacteria; o__Rhodobacterales; f__Rhodobacteraceae; g__Rubellimicrobium; s__ |
| OTU_339 | 3 | 0 | 0 | 0 | 0 | 0 | 0 | 0 | 0 | 0 | 0 | 0 | k__Bacteria; p__Bacteroidetes; c__Cytophagia; o__Cytophagales; f__Cytophagaceae; g__Pontibacter; s__ |
| OTU_340 | 0 | 1 | 2 | 0 | 0 | 0 | 0 | 0 | 0 | 0 | 0 | 0 | k__Bacteria; p__Firmicutes; c__Bacilli; o__Lactobacillales; f__Streptococcaceae; g__Streptococcus; s__anginosus |
| OTU_341 | 2 | 0 | 0 | 0 | 0 | 0 | 0 | 0 | 0 | 0 | 0 | 0 | k__Bacteria; p__Chloroflexi; c__Thermomicrobia; o__; f__; g__; s__ |
| OTU_342 | 1 | 1 | 1 | 0 | 0 | 0 | 0 | 0 | 0 | 1 | 0 | 0 | k__Bacteria; p__Cyanobacteria; c__Oscillatoriophycideae; o__Chroococcales; f__Xenococcaceae; g__; s__ |
| OTU_343 | 1 | 1 | 0 | 0 | 0 | 0 | 0 | 1 | 0 | 0 | 0 | 0 | k__Bacteria; p__Actinobacteria; c__Actinobacteria; o__Actinomycetales; f__Ruaniaceae; g__; s__ |
| OTU_344 | 2 | 0 | 2 | 0 | 0 | 0 | 1 | 0 | 0 | 0 | 0 | 0 | k__Bacteria; p__Firmicutes; c__Clostridia; o__Clostridiales; f__Veillonellaceae; g__Veillonella; s__dispar |
| OTU_345 | 0 | 0 | 2 | 0 | 0 | 0 | 0 | 0 | 0 | 0 | 0 | 0 | k__Bacteria; p__Bacteroidetes; c__Cytophagia; o__Cytophagales; f__Cytophagaceae; g__Larkinella; s__ |
| OTU_346 | 1 | 2 | 0 | 0 | 0 | 0 | 0 | 1 | 0 | 0 | 0 | 0 | k__Bacteria; p__Verrucomicrobia; c__Verruco-5; o__LD1-PB3; f__; g__; s__ |
| OTU_347 | 5 | 3 | 2 | 0 | 1 | 1 | 0 | 0 | 0 | 3 | 0 | 1 | k__Bacteria; p__Proteobacteria; c__Alphaproteobacteria; o__Rhodobacterales; f__Rhodobacteraceae; g__Amaricoccus; s__ |
| OTU_348 | 0 | 1 | 1 | 0 | 0 | 0 | 0 | 0 | 0 | 0 | 0 | 0 | k__Bacteria; p__[Thermi]; c__Deinococci; o__Deinococcales; f__Deinococcaceae; g__Deinococcus; s__ |
| OTU_349 | 1 | 1 | 1 | 1 | 0 | 0 | 0 | 0 | 0 | 1 | 0 | 0 | k__Bacteria; p__Bacteroidetes; c__Bacteroidia; o__Bacteroidales; f__Porphyromonadaceae; g__; s__ |
| OTU_350 | 1 | 2 | 0 | 1 | 0 | 0 | 0 | 0 | 0 | 1 | 0 | 0 | k__Bacteria; p__Firmicutes; c__Clostridia; o__Clostridiales; f__Dehalobacteriaceae; g__; s__ |
| OTU_351 | 1 | 1 | 1 | 0 | 0 | 0 | 0 | 0 | 0 | 0 | 0 | 0 | k__Bacteria; p__Firmicutes; c__Clostridia; o__Clostridiales; f__Lachnospiraceae |
| OTU_352 | 6 | 2 | 4 | 0 | 0 | 0 | 0 | 1 | 0 | 0 | 0 | 0 | k__Bacteria; p__Proteobacteria; c__Alphaproteobacteria; o__Sphingomonadales; f__Sphingomonadaceae; g__Kaistobacter; s__ |
| OTU_353 | 3 | 0 | 1 | 0 | 0 | 0 | 0 | 0 | 0 | 0 | 0 | 0 | k__Bacteria; p__Planctomycetes; c__Planctomycetia; o__Pirellulales; f__Pirellulaceae; g__; s__ |
| OTU_354 | 2 | 1 | 2 | 0 | 0 | 0 | 0 | 0 | 0 | 0 | 2 | 0 | k__Bacteria; p__Acidobacteria; c__[Chloracidobacteria]; o__RB41; f__; g__; s__ |
| OTU_355 | 0 | 0 | 0 | 0 | 0 | 0 | 0 | 0 | 1 | 0 | 0 | 1 | k__Bacteria; p__Firmicutes; c__Clostridia; o__Clostridiales; f__[Mogibacteriaceae]; g__; s__ |
| OTU_356 | 2 | 0 | 0 | 0 | 0 | 0 | 0 | 0 | 0 | 0 | 0 | 0 | k__Bacteria; p__Planctomycetes; c__Planctomycetia; o__Gemmatales; f__Gemmataceae; g__; s__ |
| OTU_357 | 0 | 0 | 0 | 0 | 2 | 0 | 0 | 0 | 0 | 0 | 0 | 0 | k__Bacteria; p__Bacteroidetes; c__Sphingobacteriia; o__Sphingobacteriales; f__; g__; s__ |
| OTU_358 | 1 | 0 | 0 | 0 | 0 | 0 | 0 | 2 | 0 | 0 | 1 | 0 | k__Bacteria; p__Actinobacteria; c__Actinobacteria; o__Actinomycetales; f__Yaniellaceae; g__Yaniella; s__ |
| OTU_359 | 1 | 0 | 0 | 2 | 0 | 0 | 0 | 1 | 1 | 1 | 0 | 0 | k__Bacteria; p__Firmicutes; c__Clostridia; o__Clostridiales; f__Clostridiaceae; g__Clostridium; s__ |
| OTU_360 | 0 | 0 | 1 | 0 | 0 | 0 | 0 | 0 | 0 | 1 | 0 | 0 | k__Bacteria; p__Firmicutes; c__Clostridia; o__Clostridiales; f__Lachnospiraceae; g__Blautia; s__ |
| OTU_361 | 1 | 0 | 0 | 0 | 0 | 0 | 1 | 0 | 0 | 0 | 0 | 0 | k__Bacteria; p__Proteobacteria; c__Betaproteobacteria; o__Burkholderiales; f__Alcaligenaceae; g__Oligella; s__ |
| OTU_362 | 0 | 0 | 0 | 0 | 0 | 1 | 0 | 0 | 0 | 0 | 1 | 0 | k__Bacteria; p__Firmicutes; c__Clostridia; o__Clostridiales; f__Clostridiaceae; g__Clostridium; s__ |
| OTU_363 | 0 | 0 | 0 | 0 | 0 | 0 | 0 | 0 | 0 | 2 | 0 | 0 | k__Bacteria; p__Firmicutes; c__Clostridia; o__Clostridiales; f__Lachnospiraceae; g__Epulopiscium; s__ |
| OTU_364 | 0 | 0 | 2 | 0 | 0 | 0 | 0 | 0 | 0 | 0 | 0 | 0 | k__Bacteria; p__Chlorobi; c__; o__; f__; g__; s__ |
| OTU_365 | 3 | 2 | 1 | 0 | 0 | 0 | 0 | 0 | 0 | 0 | 0 | 0 | k__Bacteria; p__Armatimonadetes; c__SJA-176; o__GAB-B06; f__; g__; s__ |
| OTU_366 | 3 | 0 | 0 | 0 | 0 | 0 | 0 | 0 | 0 | 0 | 1 | 0 | k__Bacteria; p__Proteobacteria; c__Gammaproteobacteria; o__Xanthomonadales; f__Sinobacteraceae; g__; s__ |
| OTU_367 | 1 | 0 | 1 | 0 | 0 | 0 | 0 | 0 | 0 | 0 | 0 | 0 | k__Bacteria; p__TM6; c__SJA-4; o__; f__; g__; s__ |
| OTU_368 | 3 | 4 | 1 | 0 | 0 | 0 | 1 | 0 | 0 | 0 | 0 | 0 | k__Bacteria; p__Thermotogae; c__Thermotogae; o__Thermotogales; f__Thermotogaceae; g__Kosmotoga; s__mrcj |
| OTU_369 | 0 | 2 | 1 | 0 | 0 | 0 | 0 | 0 | 0 | 0 | 0 | 0 | k__Bacteria; p__Actinobacteria; c__Actinobacteria; o__Actinomycetales; f__Intrasporangiaceae; g__; s__ |
| OTU_370 | 0 | 3 | 0 | 0 | 0 | 0 | 0 | 0 | 0 | 0 | 0 | 0 | k__Bacteria; p__Bacteroidetes; c__Bacteroidia; o__Bacteroidales; f__Bacteroidaceae; g__Bacteroides; s__ |
| OTU_371 | 1 | 0 | 0 | 0 | 0 | 0 | 1 | 0 | 0 | 0 | 0 | 0 | k__Bacteria; p__Bacteroidetes; c__Bacteroidia; o__Bacteroidales; f__; g__; s__ |
| OTU_372 | 1 | 1 | 2 | 2 | 1 | 3 | 0 | 1 | 0 | 0 | 0 | 0 | k__Bacteria; p__Firmicutes; c__Clostridia; o__Clostridiales; f__Syntrophomonadaceae; g__Syntrophomonas; s__ |
| OTU_373 | 2 | 0 | 1 | 0 | 0 | 0 | 0 | 0 | 0 | 0 | 1 | 0 | k__Bacteria; p__Actinobacteria; c__Acidimicrobiia; o__Acidimicrobiales; f__; g__; s__ |
| OTU_374 | 1 | 5 | 0 | 0 | 0 | 0 | 0 | 0 | 0 | 0 | 0 | 0 | k__Bacteria; p__Proteobacteria; c__Alphaproteobacteria; o__Rhizobiales; f__Methylobacteriaceae; g__Methylobacterium; s__adhaesivum |
